# Nature’s impact on human health and wellbeing: the scale matters

**DOI:** 10.3389/fpubh.2025.1563340

**Published:** 2025-03-10

**Authors:** Stefan Zerbe, Hannah-Lea Schmid, Claudia Hornberg, Julius Freymüller, Timothy Mc Call

**Affiliations:** ^1^Faculty of Agricultural, Environmental and Food Sciences, Free University of Bozen-Bolzano, Bozen, Italy; ^2^Institute of Geography, University of Hildesheim, Hildesheim, Germany; ^3^Medical School OWL, Sustainable Environmental Health Sciences Bielefeld University, Bielefeld, Germany

**Keywords:** biophilia, exposure measurements, greenspace, landscape, therapeutic landscapes

## Abstract

Contact with nature can have a significant influence on human physical and mental health and wellbeing. As such, various concepts and theories as well as therapeutic approaches have been developed. The term “nature,” however, covers a broad range of size and scales, ranging from individuals or small groups of animals or plants, certain ecosystems toward landscapes. The purpose of this paper is to differentiate concepts, theories, and therapy forms according to the scales of nature. We base our conceptional approach on the biological/ecological scales of species/individuals, ecosystems/land-use types, and landscapes. Based on a review, we differentiate the current state of the utilization of greenspace exposure measurements and measures assessing mental health according to these scales. We argue that a clear differentiation of biological and ecological scales provides a better understanding of the impact of nature with its components, characteristics, and dynamics on human health and wellbeing. Our paper also supports further inter- and transdisciplinary research as well as methodological approaches with regard to environment and health, such as environmental public health.

## 1 Introduction

With both increasing urbanization worldwide and land-use change, there is a growing disconnection of humans from nature (1, 2). However, a number of studies have found evidence for the positive effects of contact to nature on physical and mental health and wellbeing [e.g., (3–5)]. The term “nature” covers a broad range of size and scales, ranging from potted plants (6), individuals or small groups of animals or plants (7), a garden (8) or zoological gardens (9), to the wilderness of a national park (10) or highly transformed urban nature (11). In addition to the visible nature which addresses visual perception, there are multiple other characteristics of nature which are perceived by tactile, olfactory, auditory, and gustatory senses, as well as the dynamics of nature such as, for example, the seasons of the year (12–14). The exposure to nature can be direct or indirect, active or passive, incidental or intentional, as well as real or virtual (15, 16). Additionally, exposure is related to proximity, likelihood, and duration of nature contact (12).

The more it has become evident that contact of humans with nature has a positive effect on health and wellbeing, the more concepts (e.g., biophilia), theories (e.g., attention restoration theory), and therapeutic approaches with nature (e.g., animal-assisted therapy) have emerged or are being developed. They have been and are being developed in various scientific disciplines both, within the natural sciences (e.g., biophilia) and the social sciences (e.g., restorative environments). However, there is an increasing trend to use well-defined terminology and concepts out of contexts. This might be due to a lack of fitting terms in the respective discipline and/or in an effort to bridge certain disciplines. This partly leads to diluting the meaning of the terms or developing hybrid concepts which lack clear definitions. In environmental science and restoration ecology, for example, “ecosystem health” [e.g., (17)] is such a hybrid concept (18) which tries to merge the ecological concept of “ecosystem” with the concept of “health” from medicine and the social sciences.

**Figure 1:**
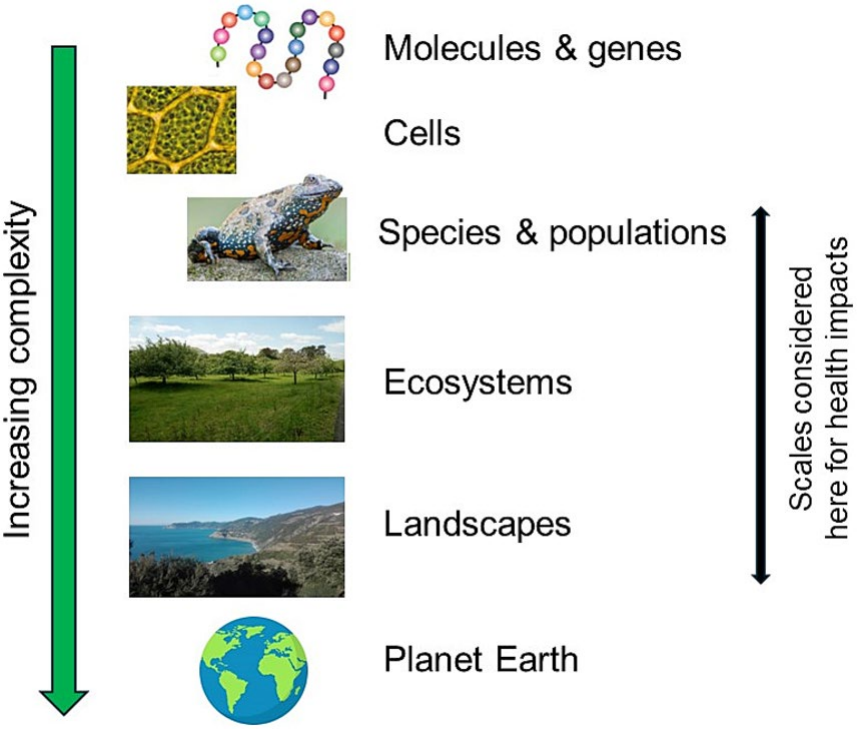
Hierarchical organization of biological structures from atoms, molecules, and genes toward Planet Earth (adapted from Sadava et al. (98) and simplified), also representing the scales of nature. Here, we focus on the scales from species, populations, and individuals to the scales of ecosystems and landscapes.

The distinction between the biological and ecological scales of species and individuals, ecosystems/land-use types, and landscapes has become less clear over time and has grown to include a large variety of settings. An example is the concept of “therapeutic landscapes” as discussed by Taheri et al. (19) which reveals the range of addressed “landscapes” from a garden to a desert [see also (20)]. This holds also true for “green” or “greenness,” often not clearly classified and differentiated (21). As such, scales of nature which are well defined in ecology and landscape ecology (Figure 1) have become mixed up, leading to an unclarity of certain concepts. Accordingly, the scales of biological organisms, ecosystems, and landscapes are no longer separated. Furthermore, many other concepts do not include information on the intended scale they pertain to, as well as empirical research that often fails to define the scales investigated (22, 23). When scale is addressed in research regarding nature and health, the focus is mostly the spatial scale from the human point of reference (24) without consideration of the biological/ecological scales [cp. (25)]. Although, this is important for investigating the exposure and experience, the first step is to define the type and scale of the “nature” in question. In fact, it has been shown that different types of nature at different scales can have different links to mental health (26).

Therefore, the aim of this paper is to address the relevance of scales when using the concepts of nature’s influence on human health and wellbeing. We base the scales of nature on the well-established biological and ecological foundations. Additionally, we analyze how the different scales are addressed in theory and research. Regarding current research, we used data gathered in the process of a scoping review (15) investigating the connection of greenspace and mental health methods to inform our findings. With this, we want to contribute to a clearer differentiation of such concepts based on the various scales of nature, the natural environment, and the elements of nature. Accordingly, (1) we define the scales of nature based on biological and ecological principles, respectively. Then, (2) we relate these scales of nature to concepts and therapies with regard to human health and wellbeing. (3) Based on a previously published scoping review, we explore the utilization of greenspace exposure measurements and measures assessing mental health according to the ecological scales and their frequency of use in order to identify common patterns and research gaps. With this conceptual approach, we hope to provide guidance for defining and differentiating “nature” based on ecological concepts, also for empirical research, especially from other disciplines. In turn, we hope that this will support further inter- and transdisciplinary research with regard to environment and health, such as environmental public health.

## 2 Methodology

**Figure 2:**
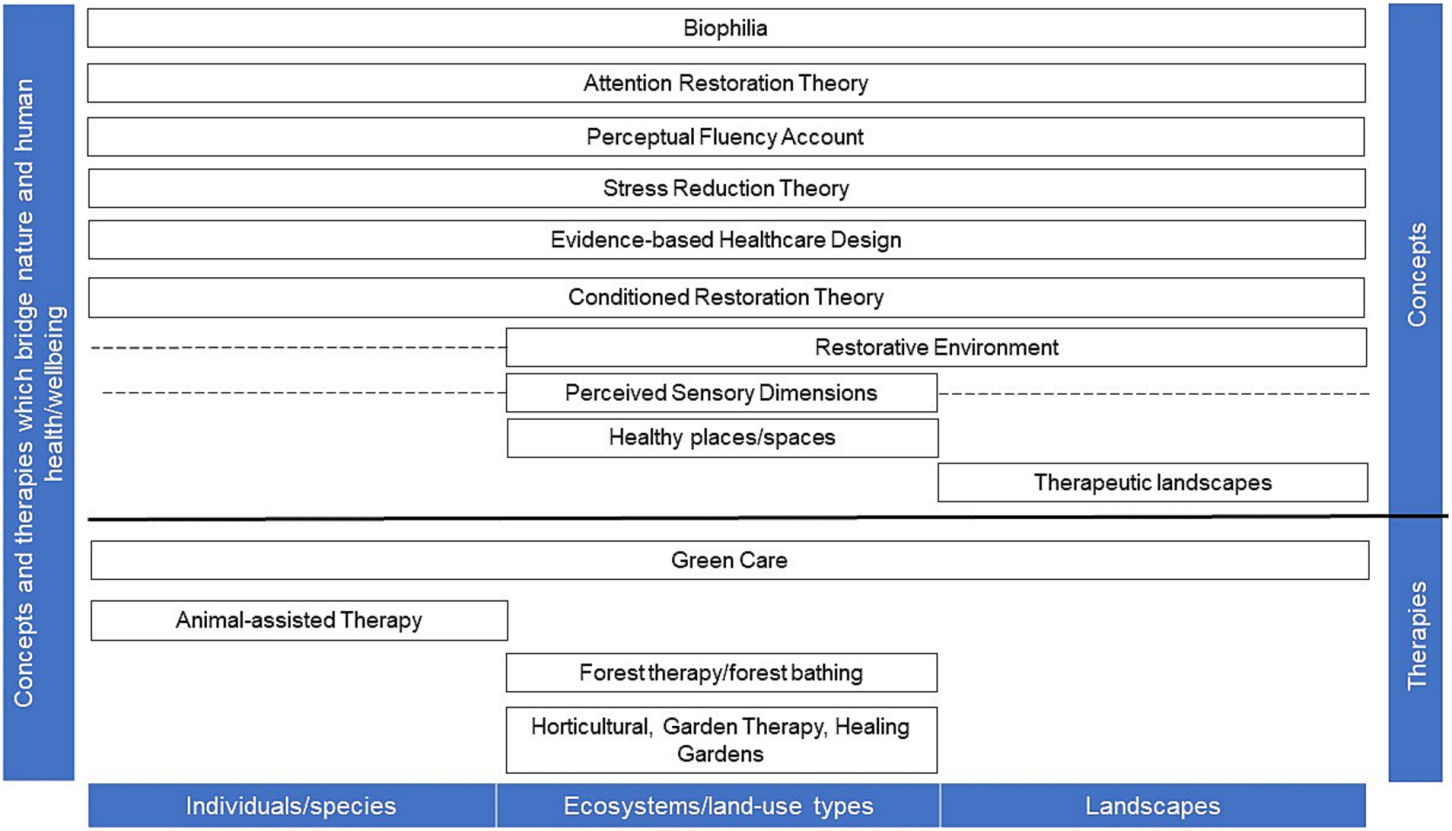
Concepts which bridge human health and wellbeing or therapies which make use of nature or natural elements related to the biological/ecological scales, ranging from individuals/species over ecosystems and land-use types to landscapes; - - - = can potentially be extended to other scales.

A multidisciplinary group was formed from two European universities (Bielefeld University and Free University of Bozen-Bolzano) with the aim of bringing together expertise in landscape ecology, urban ecology, environmental health, clinical medicine, and psychology. The group examined published reviews, as well as primary research reports, focusing on key theories, concepts, and therapeutic approaches of the nature and health nexus. Iterative discussions and consensus-building were then used to link these theories to the scales of nature. For the differentiation of the scales of nature, we refer to common approaches in biology and ecology, focusing on the scales of species/individuals, ecosystems and land-use types as well as landscapes (Figure 1; Table 1). We state examples how these three scales impact human health and give an overview on concepts and therapies related to nature and human health which we categorize according to the scales of nature (Figure 2; Table 2).

**Table 1:** Definition of the biological and ecological scales regarding species, ecosystems, and landscapes (cp. Figure 1) with selected key references.

Biological/ecological scale	Definition	Selected references	Examples which might refer to human health and wellbeing
Species	A group of organisms that can reproduce with one another in nature and produce fertile offspring. In fieldwork and for practical purposes such as, e.g., nature conservation and landscape architecture, so-called morphospecies are differentiated, taxonomically systematized, and termed, based on the morphological characters.	De Queiroz (99), Derraik et al. (100)	Trees, shrubs, herbs, grasses, horses, dogs, cats, birds, insects, reptiles
Ecosystem and land-use type	“The term ecosystem is used to denote the biological community together with the abiotic environment in which it is set. Thus, ecosystems normally include primary producers, decomposers and detritivores, a pool of dead organic matter, herbivores, carnivores and parasites plus the physicochemical environment that provides the living conditions […]”	Begon and Townsend (101)	Forest, pastureland, managed grassland, reed stand, heathland, arable land
Landscape	“Spatially heterogeneous areas characterized by a mosaic of patches that differ in size, shape, contents, and history”; they range from relatively natural terrestrial and aquatic systems such as forests, grasslands, and lakes to human-dominated environments including agricultural and urban settings	Wu (102); see also, Council of Europe (103) and Zerbe (43)	(Traditional) Cultural landscapes such as, e.g., terraced landscape, riverscape, lakescape, seascape, monastic landscapes, landscape parks as well as urban landscapes

**Table 2:** Concepts of healing nature and therapy forms taking benefit of nature and natural elements (in alphabetic order) with selected references such as the introduction of the concept or review papers.

Concept of healing nature and forms of therapies with nature and natural elements	Selected references
Concepts and theories	
Attention Restoration Theory (ART)	Kaplan and Kaplan (54)
Biophilia	Wilson (53)
Conditioned Restoration Theory (CRT)	Egner et al. (104)
Evidence-based Healthcare Design	Ulrich et al. (105)
Healthy places/spaces	Bell et al. (20)
Perceived Sensory Dimensions	Grahn and Stigsdotter (106), Schmid et al. (57)
Perceptual Fluency Account	Joye and Van der Berg (108)
Restorative Environment	Hartig (107), Joye and van den Berg (108)
Stress Reduction Theory	Luo and Jiang (109), Ulrich (110), Ulrich et al. (111)
Therapeutic Landscapes	Gesler (61)
Therapies with nature and natural elements	
Animal-assisted Therapy	Kamioka et al. (112)
Forest Therapy (incl. “Forest bathing”/*shinrin-yoku*)	Wen et al. (40), Kim and Shin (113)
Green care	Cutcliffe and Travale (114)
Horticultural, Garden Therapy, Healing Gardens	Cooper-Marcus and Barnes (35), Stigsdotter and Grahn (115), Clatworthy et al. (39), Kamioka et al. (116), Cipriani et al. (117), Dushkova and Ignatieva (36)

For the nature scales, see Figure 2.

**Figure 3:**
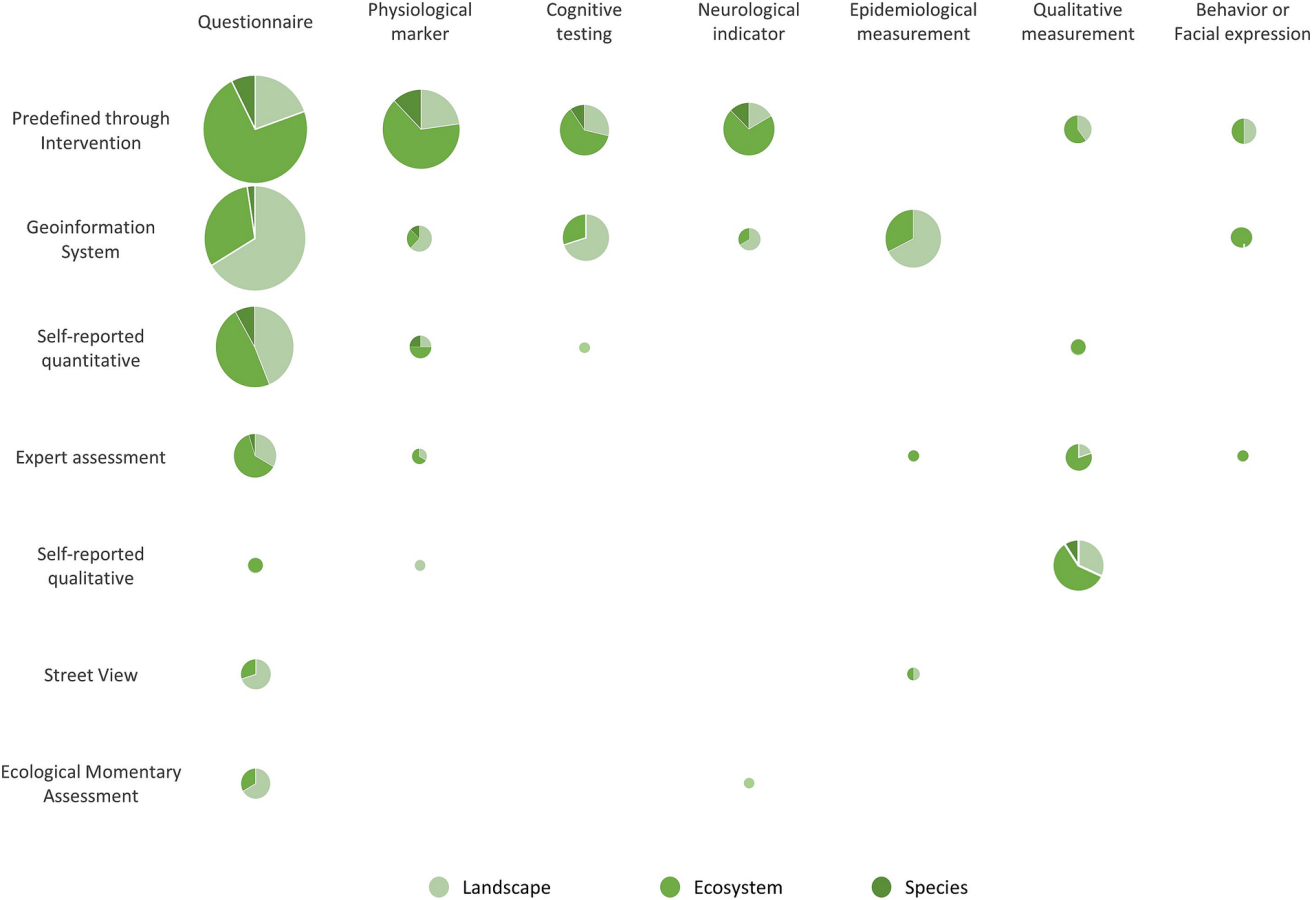
Utilization of greenspace exposure measurements and measures assessing mental health according to the ecological scales species, ecosystems, and landscapes with relative proportion and frequency of use (size of circles).

In a next step, we used data from a previously published scoping review (15) and analyzed which combinations of methods are employed at which scale in current research. The information regarding greenspace and mental health research, in particular, is based on data gathered in the context of this scoping review which focused on current methodologies of greenspace exposure and mental health research. Within this scoping review, we screened and extracted the information of 338 studies (references in Supplementary Table 1) regarding the scales and types of greenspaces, mental health outcomes, and measurements of greenspace exposure and mental health. The different categories of methods regarding greenspace and mental health in research were iteratively generated from the analyzed studies and described in the scoping review (15). Further information regarding the methodology, e.g., the screening process of the scoping review can be found in (15). We re-analyzed the data according to the three scales to identify patterns as well as potential research gaps. The biological/ecological scales used in the analysis are based on the aforementioned approaches which were identified in the existing literature. From this, we derive the up-to-date counts of utilization of greenspace exposure measurements and measures assessing mental health according to the biological/ecological scales. We visualize the distribution of the green space and mental health methods according to the scales in a bubble grid (Figure 3). The size of the bubbles is indicative of the frequency with which a specific combination of methods was utilized in comparison to other combinations. The pie charts and colors illustrate the scales and the ratio between the scales at which these combinations were employed.

## 3 Results

### 3.1 Scales in biology, ecology, and landscape ecology

Biology, ecology, and landscape ecology provide definitions of three main scales, ranging from species (and individuals of species) over ecosystems/land-use types to landscapes. These scales are defined in Table 1. In practices such as habitat (= biotope) mapping, nature conservation, ecosystem restoration or landscape planning, ecosystems often are referred to as land-use types, such as forests, grassland, heathland or arable land [e.g., (18, 27)]. The latter applies also to studies on the impact of nature and green on human health and wellbeing.

The biological and ecological differentiation of scales from molecules toward the planet (cp. Figure 1) do not necessarily correlate with spatial scales. Following the definition of a species’ habitat by Hall et al. (28) as “an area’s ability to provide resources for population persistence,” the ecosystem scale might be addressed. A monospecific reed stand [*Phragmites australis* (Cav.) Trin. Ex Steud.], for example, can cover many hectares or square kilometers, respectively, and thus, represent a wetland on the ecosystem or even landscape scale [e.g., (29)]. The Taiga of the northern hemisphere, dominated by Norway spruce (*Picea abies* L.) spans over thousands of square kilometers. Additionally, the same species has been afforested in many regions in Central Europe and thus, shaping whole mountain landscapes such as, e.g., the Thuringian forest or the Sauerland in Germany (18).

### 3.2 The various dimensions and scales of nature, and the influence on human health and wellbeing

In the following, selected examples of studies are presented in which the effect of nature and natural elements in its various dimensions and scales on human health and wellbeing are addressed. Cox et al. (30), for example, explored how individual urban trees vary in their contribution to indirect nature experiences in a human population, thus supporting urban design and planning toward green health interventions. Similarly, Zhao et al. (31) investigated the visual preference of trees by focusing on the effects of tree attributes and seasons. Finally, individuals of certain animals are often part of particular therapies such as animal-assisted interventions. Accordingly, horses (32), dogs (33), and cats (34), for example, are employed to promote human health or assist recovery from mental or physical diseases.

Ecosystems and land-use types are addressed in human health concepts, particular therapies, and health interventions. As such, healing gardens (35–37) or therapeutic gardens (38) can contribute to mental health [see also (39) on gardening-based mental health interventions]. Forests can relieve from stress, what has been coined as forest bathing (40). Emerging from Japan as “Shinrin-Yoku,” empirical research elucidates the physiological and psychological effects of forest bathing (41, 42).

The various aspects of green care can also involve the ecosystem and land-use type scale [e.g., Cutcliff et al.; for an overview, see (43)]. If farms and small-scale living facilities (44) or agricultural land (45) are related to human health or integrated into therapies, the scale of land-use types is addressed. Finally, and more general “healthy places” are a subject of health geography (20). By putting the anthropocentric ecosystem service concept [cp. (46)] to the practice of human health, Bratman et al. (12) show how ecosystem service assessments can be expanded to include mental health, and provide a heuristic, conceptual model for doing so. Often, the scale of ecosystems and land-use types is also addressed if species richness (diversity with its various indices) is related to human health [e.g., (47); see also (48)].

The benefits of landscapes to human health are explored by Opdam (49), and, in this context referred to as landscape services. Menatti and Casado da Rocha (50) discuss the concept of therapeutic landscapes and refer to, e.g., national parks and urban landscapes. The relationship between multifunctional landscapes and wellbeing is investigated by Fagerholm et al. (51) through measuring self-reported wellbeing across 13 rural and peri-urban sites in Europe.

While it is far from comprehensive, the brief overview above demonstrates the wide range of the scales of nature—species (with individuals), ecosystems, and landscapes—and their influence on both mental/physical health and human wellbeing. Human health concepts and therapies with nature partly refer to specific scales, partly not as the following chapter will elucidate.

### 3.3 Human health concepts and therapies with nature related to the various scales of nature

Figure 2 depicts how concepts and therapies which bridge nature and human health and wellbeing are related to the different biological and ecological scales of nature. Hereby, concepts are differentiated from various forms of therapies with nature and natural elements, respectively. In Table 2, these concepts and therapy forms are listed and selected references given. Concepts such as therapeutic landscapes literally address the landscape scale. Animal-assisted therapies, on the contrary, are based on the interaction of an animal individual or species with humans in order to facilitate recovery from diseases or health problems. Other concepts and therapy forms range over various scales.

Studies on therapies and theoretical concepts on the interaction of human health and nature frequently fail to address or define the scale of the environment in question. Furthermore, the different types of greenspace at the same scale are often inconsistently or inaccurately defined. Such limitations may impede the comparability of results and interdisciplinary understanding. Accordingly, we scanned through current methodologies of greenspace exposure and mental health research in order to extract the methods employed at the different scales and analyze how they differ (15).

### 3.4 Greenspace and mental health research—the question of scale

Based on data collected within the scoping review from Freymüller et al. (15), we could combine greenspace exposure methods and mental health measurements referring to the scales differentiated here (Figure 3). Generally, distinct greenspace measures are used at different scales, while the mental health measures do not show such a clear pattern. The “landscape” scale is most often assessed via GIS approaches. The scales “ecosystems” and “species” are mostly investigated via interventions. Overall, the ecosystem’s scale is the most frequent in both, research and theory. Qualitative (self-reported) mental health measures often focus on ecosystems. However, the qualitative theoretical concepts are often based on landscapes such as therapeutic landscapes. Species and natural elements are rarely directly addressed by the studies investigated. Some methods are not combined in current research (e.g., street view and physiological markers) and some combinations only employed at some scales (e.g., self-reported qualitative and questionnaire). Applying different combinations of methods at the different scales can reveal new insights as all measurements feature their own benefits and biases. Nevertheless, it should be noted that some methods can be combined better than others.

## 4 Discussion

Nature with its various dimensions, characteristics, dynamics, and scales can have a positive influence on human health and human wellbeing. Each level and scale of nature, however, provides important components in understanding what contact with nature can and cannot do for human health (52). The ecological scales can be a useful way to describe nature in health research. We demonstrated that certain concepts and therapeutic interventions directly address the question of the scales of nature, while others do not adequately address or define it. Some concepts and therapies exhibit a high degree of specificity, relating to a single scale, while others demonstrate a greater degree of versatility, applicable to a range of scales. In this regard, concepts are often broader and therapeutic approaches more specific. The present analysis of contemporary research methodologies reveals that distinct greenspace metrics are employed at varying spatial scales. In contrast, the utilization of mental health metrics does not exhibit such a consistent pattern. Furthermore, some discordance emerges between the theoretical underpinnings and the research methods employed in relation to the scale level.

### 4.1 Concepts of healing nature

Concepts, as shown in Figure 2, often address the whole range of biological-ecological scales which means, “nature *per se*.” This, for example, holds true for biophilia which describes the evolutionary adaptation of humans to nature (53). This is supported by the attention restoration theory which explains how natural environments provide positive human health and wellbeing benefits (54, 55), regardless of the scale of nature. Other concepts cannot be easily assigned to a scale such as the concept of perceived sensory dimensions. In many studies, the perceived sensory dimension concept refers to parks, gardens, and greenspaces and thus, the ecosystem and land-use scale [e.g., (56–58)]. Although, the concept refers to ecosystem services (59) and thus to the ecosystem and land-use scale, in principle, it can be applied to the whole range of nature scales. Nevertheless, Stoltz and Grahn (58) point out that a general distinction can be made between perceived sensory dimensions requiring a larger scale (natural, serene, cohesive, and open dimension) and dimensions possible on a smaller scale (shelter, diverse, social and cultural dimension). The concepts of healthy places/spaces and therapeutic landscapes however, can be clearly assigned to the ecosystem and land-use scale and the landscape scale, respectively.

First introduced by Gesler (60, 61), the concept of therapeutic landscapes has been used to draw attention to “the complex intermingling of physical, social and symbolic processes that determine a place’s potential to positively or negatively affect health” [(62), p. 10]. Various terms and terminologies have emerged in this context (20). Consequently, these “therapeutic landscapes” include a large variety of settings (19, 63), situations, and milieus (36) as well as scales of which some are indeed landscapes and some, however, address other nature scales. Accordingly, those settings studied as “therapeutic landscapes” span from the places of pilgrimage such as Lourdes in France (64), churches such as the Basilica of Sainte Anne de-Beaupré in Quebec, Canada (65), a public library (66), a cafè (67), “healing sites” such as the Asclepian sanctuary at Epidauros in Greece (68), and “symbolic landscapes” (69), over farms (70), communal and domestic gardens (71), health camps (72), and psychiatric hospitals (73, 74) to wilderness (75–78) and urban green and blue spaces (79–81).

### 4.2 Therapies with nature and nature-based therapies

In contrast, the therapy forms with which nature or natural elements are directly or indirectly applied to promote human health and wellbeing are often clearly assigned to a certain scale. This makes sense, given that interventions have to be more concrete than theories. The species scale (with its individuals of plants and animals) offers direct interaction and responsiveness which, for example, is applied within animal-assisted therapies. The ecosystem scale, encompassing land-use types in cultural landscapes, has been increasingly addressed in environment-human health research, and its implications for practice with the introduction of the ecosystem services’ concept [e.g., (82–84)]. However, the ecosystem and land-use type scale becomes more complex with its components and dimensions. This has been addressed by Lovell et al. (85) with regard to community gardening. Accordingly, the participation in the gardening activities may improve wellbeing through social contact and culturally valued activities, as well as through healthy food production.

Since the complexity increases with the landscape scale, there is no specific therapy approach yet focusing only on landscapes (Figure 2). This might be the reason for the comparably low number of studies on the impact of therapy forms employing landscapes compared to the impact of organisms and ecosystems. Accordingly, studies often are qualitative, applying interview approaches and field observations [e.g., (22, 86, 87)]; only few studies are quantitative [e.g., (88, 89)]. Given these complex conditions it is difficult to determine the effects of the landscape, e.g., the relation between the wilderness in wilderness-interventions and the therapeutic outcomes, which many studies do not directly engage with (90).

### 4.3 The complexity of nature-health interaction regarding scales

**Figure 4:**
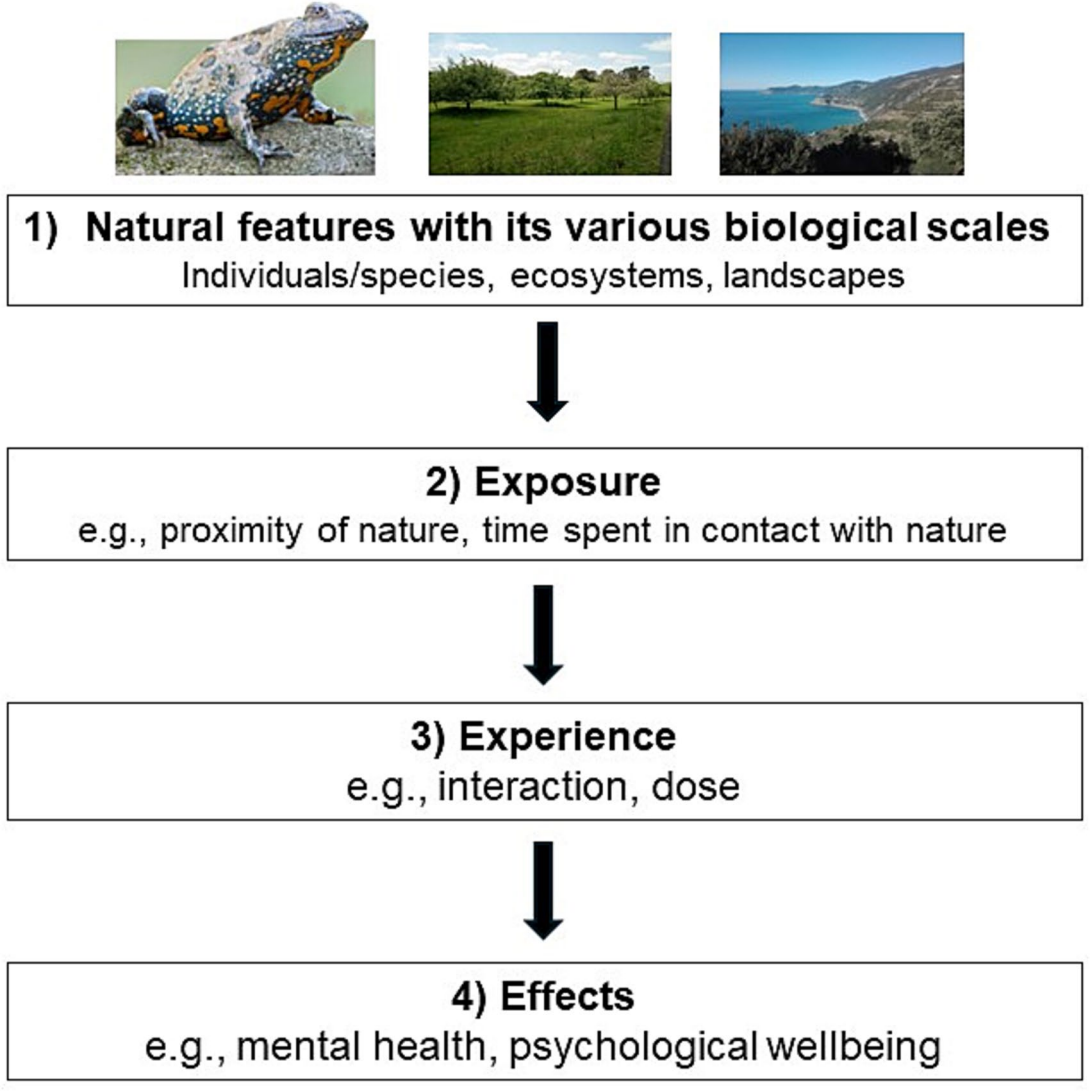
Based on the conceptual model for mental health from Bratman et al. (12), we highlight the differentiation of nature according to its different biological/ecological scales as a first and crucial step to investigate the effects of nature on human health and wellbeing.

These scales discussed here do not inform per se about the effect of nature or natural elements on human health. However, it is a first and crucial step for studying the effects of nature on human health and wellbeing (Figure 4). Accordingly, by establishing a coherent definition of biological/ecological scales can help to reveal possible differences in their effects. This is particularly relevant due to the multitude of pathways that nature such as greenspaces has on human health (91, 52). After having differentiated these scales, exposures, experiences, effects and mechanisms can be observed and measured with greater accuracy and comparability in future research.

Landscape and human health impact might not be investigated in its complexity. In their literature review, Velarde et al. (23), revealed that in studies comparing the health outcomes of visual exposure to different categories of landscapes, the categories compared were generally very coarse. They conclude that “these coarse categories clearly fail to reflect the vast variety of landscapes and landscape elements that are important in defining the character of […] landscapes” [(23), p. 208].

Besides exposure (e.g., proximity to nature, time spent in contact with nature) and experience [e.g., interaction, dose; (12)], Bratman et al. (92) point out in their review that both the scale and the different types are essential to understand the underlying psychological mechanisms for human health. The authors conclude that “at a minimum, it would be most informative were the research to specify the types of environments used in experiments in some detail, using modern quantitative methods at multiple scales “[(92), p.120]. Accordingly, this would lead to a more coherent set of postulates about which particular aspects of nature may have impacts on human health and wellbeing and what the causal pathways are for these effects. Furthermore, clear and consistent definitions of shared concepts allow more fruitful inter- and transdisciplinary research to develop (15). This will help to reveal approaches in future research that enhance beneficial outcomes for human health and wellbeing alongside nature conservation or restoration.

Other concepts, not analyzed here, are also addressing the effect of nature on human health and wellbeing at different scales. However, these concepts are often derived from existing concepts such as, e.g., “nature connectedness” as the exposure to natural environments which should have a positive impact on health and wellbeing (93). Accordingly, this concept is very similar to the theoretical framework of biophilia. The “one health” concept is an overarching framework which is “an integrated, unifying approach to balance and optimize the health of people, animals and the environment” (94). This approach is aiming at the mobilization of multiple sectors, disciplines, and communities at varying levels of society to cooperate and thus has a transdisciplinary character [see also (95)]. Similarly, “planetary health” is a transdisciplinary field and social movement which addresses human health and all life on Earth. This concept is “based on the understanding that human health and human civilization depend on flourishing natural systems and the wise stewardship of those natural systems” [(96), p. 1974]. The EcoHealth concept focuses on the interactions between the ecological and socio-economic dimensions of a given situation, and their influence on human health. Furthermore, it addresses how people use or impact ecosystems, the implications for the quality of ecosystems, the provision of ecosystem services, and sustainability (97). As one approach to mitigate negative impacts of degraded environments on human health and wellbeing, “nature-based solutions” can be considered (36). Particularly in urban environments this means the restoration of nature at all scales, from single natural elements toward landscape settings.

## 5 Conclusion

The different ecological scales are addressed using different methods and covered in different concepts and theories, respectively. Empirically, there is a clear focus on the ecosystem scale, particularly through interventions. Concepts on nature and health often comprise the whole range of ecological scales, however the scale is often not clearly stated. We would assign the concept of therapeutic landscapes to the landscape scale; however, it is often used to investigate ecosystems or species. Overall, landscapes are often assessed quantitatively through GIS methods, the therapeutic landscapes concept however has a qualitative focus. The therapeutic approaches are more clearly assigned to specific scales, with an emphasis on the species scale. In contrast, species are addressed the least in current research on greenspace and health. At the landscape level, no therapies were identified. Our study shows that increased attention to types and scales of nature is needed in both, practical research and theory. Established ecological scales can provide a common basis for interdisciplinary research and improve comparability. This will elucidate the potential differences in the impact of the diverse forms and dimensions of nature on human health. Particularly, for interdisciplinary studies which integrate (landscape) ecology and public health or medicine the differentiation of biological/ecological scales might support clearer understanding and designs of studies and their implications for practice.

Future research should focus on documenting effect sizes at the clearly defined relevant scale, for given outcomes of interest including underlying theories and concepts. In addition, a corresponding central data repository containing multiple studies or meta-analyses could be of interest to researchers and practitioners.

## Data Availability

The original contributions presented in the study are included in the article/Supplementary material, further inquiries can be directed to the corresponding author.
