# Supplementary material for: Nature’s impact on human health and wellbeing: the scale matters
Source: Front Public Health. 2025 Mar 10;13:1563340. doi: 10.3389/fpubh.2025.1563340 (PMC11931031; doi:10.3389/fpubh.2025.1563340)
Supplement: Supplementary file 1 [file Table_1.docx]

**Supplementary Table I:**

**References of the scoping review from Freymüller et al. (2024) as a basis for the analysis and results presented in Fig. 3.**

| **Author** | **Year** | **Title** | **DOI** |
| --- | --- | --- | --- |
| Abdullah, A.Y.M.; Law, J.; Butt, Z. A.; Perlman, C. M. | 2021 | Understanding the Differential Impact of Vegetation Measures on Modeling the Association between Vegetation and Psychotic and Non-Psychotic Disorders in Toronto, Canada | 10.3390/ijerph18094713 |
| Aerts, R.; Stas, M.; Vanlessen, N.; Hendrickx, M.; Bruffaerts, N.; Hoebeke, L.; Dendoncker, N.; Dujardin, S.; Saenen, N. D.; van Nieuwenhuyse, A.; Aerts, J. M.; van Orshoven, J.; Nawrot, T. S.; Somers, B. | 2020 | Residential green space and seasonal distress in a cohort of tree pollen allergy patients | 10.1016/j.ijheh.2019.10.004 |
| Aerts, R.; Vanlessen, N.; Dujardin, S.; Nemery, B.; van Nieuwenhuyse, A.; Bauwelinck, M.; Casas, L.; Demoury, C.; Plusquin, M.; Nawrot, T. S. | 2022 | Residential green space and mental health-related prescription medication sales: An ecological study in Belgium | 10.1016/j.envres.2022.113056 |
| Afrad, A.; Kawazoe, Y. | 2020 | Can interaction with informal urban green space reduce depression levels? An analysis of potted street gardens in Tangier, Morocco | 10.1016/j.puhe.2020.06.034 |
| Akpinar, A. | 2021 | How perceived sensory dimensions of urban green spaces are associated with teenagers' perceived restoration, stress, and mental health? | 10.1016/j.lurbplan.2021.104185 |
| Aliyas, Z. | 2021 | Physical, mental, and physiological health benefits of green and blue outdoor spaces among elderly people | 10.1080/09603123.2019.1681379 |
| Alyan, E.; Combe, T.; Rambli, D. R.A.; Sulaiman, S.; Merienne, F.; Diyana, N. | 2021 | The Influence of Virtual Forest Walk on Physiological and Psychological Responses | 10.3390/ijerph182111420 |
| Ameli, R.; Skeath, P.; Abraham, P. A.; Panahi, S.; Kazman, J. B.; Foote, F.; Deuster, P. A.; Ahmad, N.; Berger, A. | 2021 | A nature-based health intervention at a military healthcare center: a randomized, controlled, cross-over study | 10.7717/peerj.10519 |
| An, Congying; Liu, Jinglan; Liu, Qiaohui; Liu, Yuqi; Fan, Xiaoli; Hu, Yishen | 2022 | How Perceived Sensory Dimensions of Forest Park Are Associated with Stress Restoration in Beijing? | 10.3390/ijerph19020883 |
| Arnberger, A.; Eder, R.; Allex, B.; Ebenberger, M.; Hutter, H. P.; Wallner, P.; Bauer, N.; Zaller, J. G.; Frank, T. | 2018 | Health-Related Effects of Short Stays at Mountain Meadows, a River and an Urban SiteResults from a Field Experiment | 10.3390/ijerph15122647 |
| Astell-Burt, T.; Feng, X. | 2020 | Greener neighbourhoods, better memory? A longitudinal study | 10.1016/j.healthplace.2020.102393 |
| Astell-Burt, T.; Feng, X. Q. | 2019 | Association of Urban Green Space With Mental Health and General Health Among Adults in Australia | 10.1001/jamanetworkopen.2019.8209 |
| Astell-Burt, T.; Navakatikyan, M. A.; Feng, X. | 2020 | Urban green space, tree canopy and 11-year risk of dementia in a cohort of 109,688 Australians | 10.1016/j.envint.2020.106102 |
| Astell-Burt, T.; Navakatikyan, M.; Eckermann, S.; Hackett, M.; Feng, X. | 2022 | Is urban green space associated with lower mental healthcare expenditure? | 10.1016/j.socscimed.2021.114503 |
| Aziz, N. A.A.; Shian, L. Y.; Mokhtar, M. D.M.; Raman, T. L.; Saikim, F. H.; Chen, W.; Nordin, N. M. | 2021 | Effectiveness of urban green space on undergraduates' stress relief in tropical city: A field experiment in Kuala Lumpur | 10.1016/j.ufug.2021.127236 |
| Baena-Extremera, A.; Garcia, J. F.; Martinez, A. C.; Martin-Perez, C. | 2021 | Sports in Natural Environment, Sports in Urban Environment: An fMRI Study about Stress and Attention/Awareness | 10.52082/jssm.2021.789 |
| Bailey, A.; Kingsley, J. | 2022 | Valuing the Benefits and Enhancing Access: Community and Allotment Gardens in Urban Melbourne, Australia | 10.3390/land11010062 |
| Bailey, A.; Kingsley, J. | 2020 | Connections in the garden: opportunities for wellbeing | 10.1080/13549839.2020.1845637 |
| Bakolis, I.; Hammoud, R.; Smythe, M.; Gibbons, J.; Davidson, N.; Tognin, S.; Mechelli, A. | 2018 | Urban Mind: Using Smartphone Technologies to Investigate the Impact of Nature on Mental-Well-Being in Real Time | 10.1093/biosci/bix149 |
| Banay, R. F.; James, P.; Hart, J. E.; Kubzansky, L. D.; Spiegelman, D.; Okereke, O. L.; Spengler, J. D.; Laden, F. | 2019 | Greenness and Depression Incidence among Older Women | 10.1289/EHP1229 |
| Barreto, P. A.; Lopes, C. S.; Da Silveira, I. H.; Faerstein, E.; Junger, W. L. | 2019 | Is living near green areas beneficial to mental health? Results of the Pro-Saude Study | 10.11606/s1518-8787.2019053001008 |
| Basu, M.; DasGupta, R.; Kumar, P.; Dhyani, S. | 2021 | Home gardens moderate the relationship between Covid-19-induced stay-at-home orders and mental distress: a case study with urban residents of India | 10.1088/2515-7620/ac2ab2 |
| Besser, L. M.; Hirsch, J.; Galvin, J. E.; Renne, J.; Park, J.; Evenson, K. R.; Kaufman, J. D.; Fitzpatrick, A. L. | 2020 | Associations between neighborhood park space and cognition in older adults vary by US location: The Multi-Ethnic Study of Atherosclerosis | 10.1016/j.healthplace.2020.102459 |
| Beute, F.; Kort, Y. A.W. de | 2018 | Stopping the Train of Thought: A Pilot Study Using an Ecological Momentary Intervention with Twice-Daily Exposure to Natural versus Urban Scenes to Lower Stress and Rumination | 10.1111/aphw.12128 |
| Bezold, C. P.; Banay, R. F.; Coull, B. A.; Hart, J. E.; James, P.; Kubzansky, L. D.; Missmer, S. A.; Laden, F. | 2018 | The Association Between Natural Environments and Depressive Symptoms in Adolescents Living in the United States | 10.1016/j.jadohealth.2017.10.008 |
| Bezold, C. P.; Banay, R. F.; Coull, B. A.; Hart, J. E.; James, P.; Kubzansky, L. D.; Missmer, S. A.; Laden, F. | 2018 | The relationship between surrounding greenness in childhood and adolescence and depressive symptoms in adolescence and early adulthood | 10.1016/j.annepidem.2018.01.009 |
| Bielinis, E.; Omelan, A.; Boiko, S.; Bielinis, L. | 2018 | The Restorative Effect of Staying in a Broad-Leaved Forest on Healthy Young Adults in Winter and Spring |  |
| Bielinis, E.; Simkin, J.; Puttonen, P.; Tyrvainen, L. | 2020 | Effect of Viewing Video Representation of the Urban Environment and Forest Environment on Mood and Level of Procrastination | 10.3390/ijerph17145109 |
| Bijnens, E. M.; Vos, S.; Verheyen, V. V.; Bruckers, L.; Covaci, A.; Henauw, S. de; Hond, E. den; Loots, I.; Nelen, V.; Plusquin, M.; Schoeters, G.; Nawrot, T. S. | 2022 | Higher surrounding green space is associated with better attention in Flemish adolescents | 10.1016/j.envint.2021.107016 |
| Birch, J.; Rishbeth, C.; Payne, [SR] | 2020 | Nature doesn't judge you - how urban nature supports young people's mental health and wellbeing in a diverse UK city | 10.1016/j.healthplace.2020.102296 |
| Bitterman, N.; Simonov, E. | 2017 | Multisensory design of pocket gardens for reducing stress and improving well-being, performance and satisfaction | 10.1080/14606925.2017.1352755 |
| Bloemsma, L. D.; Wijga, A. H.; Klompmaker, J. O.; Hoek, G.; Janssen, N. A.H.; Oldenwening, M.; Koppelman, G. H.; Lebret, E.; Brunekreef, B.; Gehring, U. | 2021 | Green space, air pollution, traffic noise and saliva cortisol in children The PIAMA study | 10.1097/EE9.0000000000000141 |
| Bloemsma, L. D.; Wijga, A. H.; Klompmaker, J. O.; Hoek, G.; Janssen, N.A.H.; Lebret, E.; Brunekreef, B.; Gehring, U. | 2022 | Green space, air pollution, traffic noise and mental wellbeing throughout adolescence: Findings from the PIAMA study | 10.1016/j.envint.2022.107197 |
| Boers, S.; Hagoort, K.; Scheepers, F.; Helbich, M. | 2018 | Does Residential Green and Blue Space Promote Recovery in Psychotic Disorders? A Cross-Sectional Study in the Province of Utrecht, The Netherlands | 10.3390/ijerph15102195 |
| Bojorquez, I.; Ojeda-Revah, L. | 2018 | Urban public parks and mental health in adult women: Mediating and moderating factors | 10.1177/0020764018795198 |
| Boll, L. M.; Khamirchi, R.; Alonso, L.; Llurba, E.; Pozo, O. J.; Miri, M.; Dadvand, P. | 2020 | Prenatal greenspace exposure and cord blood cortisol levels: A cross-sectional study in a middle-income country | 10.1016/j.envint.2020.106047 |
| Bourrier, Stefan C.; Berman, Marc G.; Enns, James T. | 2018 | Cognitive Strategies and Natural Environments Interact in Influencing Executive Function | 10.3389/fpsyg.2018.01248 |
| Brace, O.; Garrido-Cumbrera, M.; Foley, R.; Correa-Fernandez, J.; Suarez-Caceres, G.; Lafortezza, R. | 2020 | Is a View of Green Spaces from Home Associated with a Lower Risk of Anxiety and Depression? | 10.3390/ijerph17197014 |
| Brancato, G. G.; van Hedger, K.; Berman, M. G.; van Hedger, S. C. | 2022 | Simulated nature walks improve psychological well-being along a natural to urban continuum | 10.1016/j.jenvp.2022.101779 |
| Brito, Junia N. de; Pope, Zachary C.; Mitchell, Nathan R.; Schneider, Ingrid E.; Larson, Jean M.; Horton, Teresa H.; Pereira, Mark A. | 2019 | Changes in Psychological and Cognitive Outcomes after Green versus Suburban Walking: A Pilot Crossover Study | 10.3390/ijerph16162894 |
| Cassarino, M.; Tuohy, I. C.; Setti, A. | 2019 | Sometimes Nature Doesn't Work: Absence of Attention Restoration in Older Adults Exposed to Environmental Scenes | 10.1080/0361073X.2019.1627497 |
| Chang, D. H.F.; Jiang, B.; Wong, N. H.L.; Wong, J. J.; Webster, C.; Lee, T. M.C. | 2021 | The human posterior cingulate and the stress-response benefits of viewing green urban landscapes | 10.1016/j.neuroimage.2020.117555 |
| Chang, H. T.; Wu, C. D.; Wang, J. D.; Chen, P. S.; Su, H. J. | 2021 | Residential green space structures are associated with a lower risk of bipolar disorder: A nationwide population-based study in Taiwan | 10.1016/j.envpol.2020.115864 |
| Chang, H. T.; Wu, C. D.; Wang, J. D.; Chen, P. S.; Wang, Y. J.; Su, H. J. | 2020 | Green space structures and schizophrenia incidence in Taiwan: is there an association? | 10.1088/1748-9326/ab91e8 |
| Chang, H.-T.; Wu, C.-D.; Pan, W.-C.; Candice Lung, S.-C.; Su, H.-J. | 2019 | Association between surrounding greenness and schizophrenia: A taiwanese cohort study | 10.3390/ijerph16081415 |
| Cheng, Y. Y.; Zhang, J. G.; Wei, W.; Zhao, B. | 2021 | Effects of urban parks on residents' expressed happiness before and during the COVID-19 pandemic | 10.1016/j.lurbplan.2021.104118 |
| Cherrie, M. P.C.; Shortt, N. K.; Mitchell, R. J.; Taylor, A.M.; Redmond, P.; Thompson, C. W.; Starr, J. M.; Deary, I. J.; Pearce, J.R. | 2018 | Green space and cognitive ageing: A retrospective life course analysis in the Lothian Birth Cohort 1936 | 10.1016/j.socscimed.2017.10.038 |
| Cherrie, M.P.C.; Shortt, N. K.; Thompson, C. W.; Deary, I. J.; Pearce, J.R. | 2019 | Association between the activity space exposure to parks in childhood and adolescence and cognitive aging in later life | 10.3390/ijerph16040632 |
| Chi, D.; Aerts, R.; van Nieuwenhuyse, A.; Bauwelinck, M.; Demoury, C.; Plusquin, M.; Nawrot, T. S.; Casas, L.; Somers, B. | 2022 | Residential Exposure to Urban Trees and Medication Sales for Mood Disorders and Cardiovascular Disease in Brussels, Belgium: An Ecological Study | 10.1289/EHP9924 |
| Chiang, Y. C.; Li, D. Y. | 2019 | Metric or topological proximity? The associations among proximity to parks, the frequency of residents' visits to parks, and perceived stress | 10.1016/j.ufug.2018.12.011 |
| Chou, W. Y.; Hung, S. H. | 2021 | Cumulative Frequency of Nature Dose: How Continuous and Regular Forest Walking Improves Nature Relatedness, Restorativeness, and Learning Engagement in College Students | 10.3390/su132011370 |
| Chung, K.; Lee, D.; Park, J. Y. | 2018 | Involuntary Attention Restoration During Exposure to Mobile-Based 360 degrees Virtual Nature in Healthy Adults With Different Levels of Restorative Experience: Event-Related Potential Study | 10.2196/11152 |
| Cleary, A.; Roiko, A.; Burton, N. W.; Fielding, K. S.; Murray, Z.; Turrell, G. | 2019 | Changes in perceptions of urban green space are related to changes in psychological well-being: Cross-sectional and longitudinal study of mid-aged urban residents | 10.1016/j.healthplace.2019.102201 |
| Coldwell, D.F.; Evans, K. L. | 2018 | Visits to urban green-space and the countryside associate with different components of mental well-being and are better predictors than perceived or actual local urbanisation intensity | 10.1016/j.landurbplan.2018.02.007 |
| Colley, K.; Brown, C.; Montarzino, A. | 2017 | Understanding Knowledge Workers' Interactions With Workplace Greenspace: Open Space Use and Restoration Experiences at Urban-Fringe Business Sites | 10.1177/0013916516629194 |
| Cottagiri, S.A.; Villeneuve, P.J.; Raina, P.; Le Griffith; Rainham, D.; Dales, R.; Peters, C.E.; Ross, N.A.; Crouse, D L. | 2022 | Increased urban greenness associated with improved mental health among middle-aged and older adults of the Canadian Longitudinal Study on Aging (CLSA) | 10.1016/j.envres.2021.112587 |
| Cox, D. T.C.; Shanahan, D. F.; Hudson, H. L.; Plummer, K. E.; Siriwardena, G. M.; Fuller, R. A.; Anderson, K.; Hancock, S.; Gaston, K. J. | 2017 | Doses of Neighborhood Nature: The Benefits for Mental Health of Living with Nature | 10.1093/biosci/biw173 |
| Crous-Bou, M.; Gascon, M.; Gispert, J. D.; Cirach, M.; Sánchez-Benavides, G.; Falcon, C.; Arenaza-Urquijo, E. M.; Gotsens, X.; Fauria, K.; Sunyer, J.; Nieuwenhuijsen, M. J.; Luis Molinuevo, J. | 2020 | Impact of urban environmental exposures on cognitive performance and brain structure of healthy individuals at risk for Alzheimer's dementia | 10.1016/j.envint.2020.105546 |
| Crouse, D. L.; Pinault, L.; Christidis, T.; Lavigne, E.; Thomson, E. M.; Villeneuve, P. J. | 2021 | Residential greenness and indicators of stress and mental well-being in a Canadian national-level survey | 10.1016/j.envres.2020.110267 |
| Deng, L.; Li, X.; Luo, H.; Fu, E. K.; Ma, J.; Sun, L. X.; Huang, Z.; Cai, S. Z.; Jia, Y. | 2020 | Empirical study of landscape types, landscape elements and landscape components of the urban park promoting physiological and psychological restoration | 10.1016/j.ufug.2019.126488 |
| Di, N.; Li, S. S.; Xiang, H.; Xie, Y. Y.; Mao, Z. X.; Hou, J.; Liu, X. T.; Huo, W. Q.; Yang, B. Y.; Dong, G. H.; Wang, C. J.; Chen, G. B.; Guo, Y. M. | 2020 | Associations of Residential Greenness with Depression and Anxiety in Rural Chinese Adults | 10.1016/j.xinn.2020.100054 |
| Dipeolu, A. A.; Ibem, E. O.; Fadamiro, J. A.; Omoniyi, S. S.; Aluko, R. O. | 2021 | Influence of green infrastructure on residents' self-perceived health benefits in Lagos metropolis, Nigeria | 10.1016/j.cities.2021.103378 |
| Djeudeu, D.; Engel, M.; Jöckel, K-H; Moebus, S.; Ickstadt, K. | 2020 | Spatio-temporal analysis of the risk of depression at district-level and association with greenness based on the Heinz Nixdorf Recall Study | 10.1016/j.sste.2020.100340 |
| Douglas, J. W.A.; Evans, K. L. | 2022 | An experimental test of the impact of avian diversity on attentional benefits and enjoyment of people experiencing urban green-space | 10.1002/pan3.10279 |
| Dybvik, J. B.; Sundsford, S.; Wang, C. E.A.; Nivison, M. | 2018 | Significance of nature in a clinical setting and its perceived therapeutic value from patients' perspective | 10.1080/13642537.2018.1529690 |
| Dzhambov, A.; Hartig, T.; Markevych, I.; Tilov, B.; Dimitrova, D. | 2018 | Urban residential greenspace and mental health in youth: Different approaches to testing multiple pathways yield different conclusions | 10.1016/j.envres.2017.09.015 |
| Dzhambov, A.M. | 2018 | Residential green and blue space associated with better mental health: a pilot follow-up study in university students | 10.2478/aiht-2018-69-3166 |
| Dzhambov, A.M.; Bahchevanov, K. M.; Chompalov, K. A.; Atanassova, P. A. | 2019 | A feasibility study on the association between residential greenness and neurocognitive function in middle-aged Bulgarians | 10.2478/aiht-2019-70-3326 |
| Dzhambov, A.M.; Hartig, T.; Tilov, B.; Atanasova, V.; Makakova; Dimitrova, D. D. | 2019 | Residential greenspace is associated with mental health via intertwined capacity-building and capacity-restoring pathways | 10.1016/j.envres.2019.108708 |
| Dzhambov, A.M.; Lercher, P.; Browning, MHEM; Stoyanov, D.; Petrova, N.; Novakov, S.; Dimitrova, D. D. | 2021 | Does greenery experienced indoors and outdoors provide an escape and support mental health during the COVID-19 quarantine? | 10.1016/j.envres.2020.110420 |
| Dzhambov, A.M.; Markevych, I.; Hartig, T.; Tilov, B.; Arabadzhiev, Z.; Stoyanov, D.; Gatseva, P.; Dimitrova, D. D. | 2018 | Multiple pathways link urban green- and bluespace to mental health in young adults | 10.1016/j.envres.2018.06.004 |
| Egorov, A. I.; Griffin, S. M.; Converse, R. R.; Styles, J. N.; Sams, E. A.; Wilson, A.; Le Jackson; Wade, T. J. | 2017 | Vegetated land cover near residence is associated with reduced allostatic load and improved biomarkers of neuroendocrine, metabolic and immune functions | 10.1016/j.envres.2017.07.009 |
| Elsadek, M.; Liu, B. Y.; Lian, Z. F. | 2019 | Green facades: Their contribution to stress recovery and well-being in high-density cities | 10.1016/j.ufug.2019.126446 |
| Elsadek, M.; Liu, B. Y.; Lian, Z. F.; Xie, J. F. | 2019 | The influence of urban roadside trees and their physical environment on stress relief measures: A field experiment in Shanghai | 10.1016/j.ufug.2019.05.007 |
| Elsadek, M.; Liu, B. Y.; Xie, J. F. | 2020 | Window view and relaxation: Viewing green space from a high-rise estate improves urban dwellers' wellbeing | 10.1016/j.ufug.2020.126846 |
| Elsadek, M.; Shao, Y. H.; Liu, B. Y. | 2021 | Benefits of Indirect Contact With Nature on the Physiopsychological Well-Being of Elderly People | 10.1177/19375867211006654 |
| Engemann, K.; Pedersen, C. B.; Agerbo, E.; Arge, L.; Børglum, A. D.; Erikstrup, C.; Hertel, O.; Hougaard, D. M.; McGrath, J. J.; Mors, O.; Mortensen, P. B.; Nordentoft, M.; Sabel, C. E.; Sigsgaard, T.; Tsirogiannis, C.; Vilhjálmsson, B. J.; Werge, T.; Svenning, J.-C.; Horsdal, H. T. | 2020 | Association between Childhood Green Space, Genetic Liability, and the Incidence of Schizophrenia | 10.1093/schbul/sbaa058 |
| Engemann, K.; Pedersen, C. B.; Arge, L.; Tsirogiannis, C.; Mortensen, P. B.; Svenning, J. C. | 2019 | Residential green space in childhood is associated with lower risk of psychiatric disorders from adolescence into adulthood | 10.1073/pnas.1807504116 |
| Engemann, K.; Svenning, J. C.; Arge, L.; Brandt, J.; Bruun, M. T.; Didriksen, M.; Erikstrup, C.; Geels, C.; Hertel, O.; Horsdal, H. T.; Kaspersen, K. A.; Mikkelsen, S.; Mortensen, P. B.; Nielsen, K. R.; Ostrowski, [SR]; Pedersen, O. B.; Tsirogiannis, C.; Sabel, C. E.; Sigsgaard, T.; Ullum, H.; Pedersen, C. B. | 2021 | A life course approach to understanding associations between natural environments and mental well-being for the Danish blood donor cohort | 10.1016/j.healthplace.2021.102678 |
| Engemann, K.; Svenning, J. C.; Arge, L.; Brandt, J.; Erikstrup, C.; Geels, C.; Hertel, O.; Mortensen, P. B.; Plana-Ripoll, O.; Tsirogiannis, C.; Sabel, C. E.; Sigsgaard, T.; Pedersen, C. B. | 2020 | Associations between growing up in natural environments and subsequent psychiatric disorders in Denmark | 10.1016/j.envres.2020.109788 |
| Engemann, K.; Svenning, J. C.; Arge, L.; Brandt, J.; Geels, C.; Mortensen, P. B.; Plana-Ripoll, O.; Tsirogiannis, C.; Pedersen, C. B. | 2020 | Natural surroundings in childhood are associated with lower schizophrenia rates | 10.1016/j.schres.2019.10.012 |
| Ewert, A.; Chang, Y. | 2018 | Levels of Nature and Stress Response | 10.3390/bs8050049 |
| Feng, X. Q.; Astell-Burt, T. | 2018 | Residential green space quantity and quality and symptoms of psychological distress: a 15-year longitudinal study of 3897 women in postpartum | 10.1186/s12888-018-1926-1 |
| Feng, X. Q.; Toms, R.; Astell-Burt, T. | 2022 | The nexus between urban green space, housing type, and mental health | 10.1007/s00127-022-02266-2 |
| Fisher, J. C.; Bicknell, J. E.; Irvine, K. N.; Hayes, W. M.; Fernandes, D.; Mistry, J.; Davies, Z. G. | 2021 | Bird diversity and psychological wellbeing: A comparison of green and coastal blue space in a neotropical city | 10.1016/j.scitotenv.2021.148653 |
| Franek, M.; Petruzalek, J. | 2021 | Viewing Natural vs. Urban Images and Emotional Facial Expressions: An Exploratory Study | 10.3390/ijerph18147651 |
| Franklin, M.; Yin, X. Z.; McConnell, R.; Fruin, S. | 2020 | Association of the Built Environment With Childhood Psychosocial Stress | 10.1001/jamanetworkopen.2020.17634 |
| Gao, J.; Mancus, G. C.; Yuen, H. K.; Watson, J. H.; Lake, M. L.; Jenkins, G. R. | 2021 | Changes in cortisol and dehydroepiandrosterone levels immediately after urban park visits | 10.1080/09603123.2021.2013454 |
| Gao, T.; Song, R.; Zhu, L.; Qiu, L. | 2019 | What Characteristics of Urban Green Spaces and Recreational Activities Do Self-Reported Stressed Individuals Like? A Case Study of Baoji, China | 10.3390/ijerph16081348 |
| Gao, T.; Zhang, T.; Zhu, L.; Gao, Y. A.; Qiu, L. | 2019 | Exploring Psychophysiological Restoration and Individual Preference in the Different Environments Based on Virtual Reality | 10.3390/ijerph16173102 |
| Gascon, M.; Sanchez-Benavides, G.; Dadvand, P.; Martinez, D.; Gramunt, N.; Gotsens, X.; Cirach, M.; Vert, C.; Molinuevo, J. L.; Crous-Bou, M.; Nieuwenhuijsen, M. | 2018 | Long-term exposure to residential green and blue spaces and anxiety and depression in adults: A cross-sectional study | 10.1016/j.envres.2018.01.012 |
| Gerber, Stephan M.; Jeitziner, Marie-Madlen; Sänger, Simon D.; Knobel, Samuel E. J.; Marchal-Crespo, Laura; Müri, René M.; Schefold, Joerg C.; Jakob, Stephan M.; Nef, Tobias | 2019 | Comparing the Relaxing Effects of Different Virtual Reality Environments in the Intensive Care Unit: visualisational Study | 10.2196/15579 |
| Golding, S. E.; Gatersleben, B.; Cropley, M. | 2018 | An Experimental Exploration of the Effects of Exposure to Images of Nature on Rumination | 10.3390/ijerph15020300 |
| Goncalves, P.; Grilo, F.; Mendes, R. C.; Vierikko, K.; Elands, B.; Marques, T. A.; Santos-Reis, M. | 2021 | What's biodiversity got to do with it? Perceptions of biodiversity and restorativeness in urban parks | 10.5751/ES-12598-260325 |
| Gonzales-Inca, C.; Pentti, J.; Stenholm, S.; Suominen, S.; Vahtera, J.; Käyhkö, N. | 2022 | Residential greenness and risks of depression: Longitudinal associations with different greenness indicators and spatial scales in a Finnish population cohort | 10.1016/j.healthplace.2022.102760 |
| Gray, T.; Tracey, D.; Truong, S.; Ward, K. | 2022 | Community gardens as local learning environments in social housing contexts: participant perceptions of enhanced wellbeing and community connection | 10.1080/13549839.2022.2048255 |
| Ha, J.; Kim, H. J.; With, K. A. | 2022 | Urban green space alone is not enough: A landscape analysis linking the spatial distribution of urban green space to mental health in the city of Chicago | 10.1016/j.lurbplan.2021.104309 |
| Hadavi, S. | 2017 | Direct and Indirect Effects of the Physical Aspects of the Environment on Mental Well-Being | 10.1177/0013916516679876 |
| Hartley, K.; Perazzo, J.; Brokamp, C.; Gillespie, G. L.; Cecil, K. M.; LeMasters, G.; Yolton, K.; Ryan, P. | 2021 | Residential surrounding greenness and self-reported symptoms of anxiety and depression in adolescents | 10.1016/j.envres.2020.110628 |
| Hassan, A.; Tao, J.; Li, G.; Jiang, M. Y.; Aii, L.; Jiang, Z. H.; Liu, Z. F.; Chen, Q. B. | 2018 | Effects of Walking in Bamboo Forest and City Environments on Brainwave Activity in Young Adults | 10.1155/2018/9653857 |
| Hatala, A. R.; Njeze, C.; Morton, D.; Pearl, T.; Bird-Naytowhow, K. | 2020 | Land and nature as sources of health and resilience among Indigenous youth in an urban Canadian context: a photovoice exploration | 10.1186/s12889-020-08647-z |
| He, J. L.; Li, L.; Li, J. M. | 2022 | Generating Inclusive Health Benefits from Urban Green Spaces: An Empirical Study of Beijing Olympic Forest Park | 10.3390/buildings12040397 |
| Helbich, M.; Klein, N.; Roberts, H.; Hagedoorn, P.; Groenewegen, P. P. | 2018 | More green space is related to less antidepressant prescription rates in the Netherlands: A Bayesian geoadditive quantile regression approach | 10.1016/j.envres.2018.06.010 |
| Helbich, M.; Poppe, R.; Oberski, D.; van Emmichoven, M. Z.; Schram, R. | 2021 | Can't see the wood for the trees? An assessment of street view- and satellite-derived greenness measures in relation to mental health | 10.1016/j.lurbplan.2021.104181 |
| Helbich, M.; Yao, Y.; Liu, Y.; Zhang, J. B.; Liu, P. H.; Wang, R. Y. | 2019 | Using deep learning to examine street view green and blue spaces and their associations with geriatric depression in Beijing, China | 10.1016/j.envint.2019.02.013 |
| Henson, Philip; Pearson, John F.; Keshavan, Matcheri; Torous, John | 2020 | Impact of dynamic greenspace exposure on symptomatology in individuals with schizophrenia | 10.1371/journal.pone.0238498 |
| Herman, K.; Ciechanowski, L.; Przegalinska, A. | 2021 | Emotional Well-Being in Urban Wilderness: Assessing States of Calmness and Alertness in Informal Green Spaces (IGSs) with Muse-Portable EEG Headband | 10.3390/su13042212 |
| Herrera, R.; Markevych, I.; Berger, U.; Genuneit, J.; Gerlich, J.; Nowak, D.; Schlotz, W.; Vogelberg, C.; Mutius, E. von; Weinmayr, G.; Windstetter, D.; Weigl, M.; Heinrich, J.; Radon, K. | 2018 | Greenness and job-related chronic stress in young adults: a prospective cohort study in Germany | 10.1136/bmjopen-2018-021599 |
| Hicks, L. J.; Smith, A. C.; Ralph, B. C.W.; Smilek, D. | 2020 | Restoration of sustained attention following virtual nature exposure: Undeniable or unreliable? | 10.1016/j.jenvp.2020.101488 |
| Hidalgo, A. K. | 2021 | Mental health in winter cities: The effect of vegetation on streets | 10.1016/j.ufug.2021.127226 |
| Hofmann, M.; Young, C.; Binz, T. M.; Baumgartner; Bauer, N. | 2018 | Contact to Nature Benefits Health: Mixed Effectiveness of Different Mechanisms | 10.3390/ijerph15010031 |
| Hoj, S. B.; Paquet, C.; Caron, J.; Daniel, M. | 2021 | Relative 'greenness' and not availability of public open space buffers stressful life events and longitudinal trajectories of psychological distress | 10.1016/j.healthplace.2020.102501 |
| Houlden, V.; Weich, S.; Jarvis, S. | 2017 | A cross-sectional analysis of green space prevalence and mental wellbeing in England | 10.1186/s12889-017-4401-x |
| Huang, Q. Y.; Yang, M. Y.; Jane, H. A.; Li, S. H.; Bauer, N. | 2020 | Trees, grass, or concrete? The effects of different types of environments on stress reduction | 10.1016/j.landurbplan.2019.103654 |
| Huang, S. P.; Qi, J. D.; Li, W.; Dong, J. W.; van den Bosch, C. K. | 2021 | The Contribution to Stress Recovery and Attention Restoration Potential of Exposure to Urban Green Spaces in Low-Density Residential Areas | 10.3390/ijerph18168713 |
| Hubbard, G.; Daas, C. den; Johnston, M.; Murchie, P.; Thompson, C. W.; Dixon, D. | 2021 | Are Rurality, Area Deprivation, Access to Outside Space, and Green Space Associated with Mental Health during the COVID-19 Pandemic? A Cross Sectional Study (CHARIS-E) | 10.3390/ijerph18083869 |
| Huerta, C. M.; Utomo, A. | 2021 | Evaluating the association between urban green spaces and subjective well-being in Mexico city during the COVID-19 pandemic | 10.1016/j.healthplace.2021.102606 |
| Hunter; Gillespie, B. W.; Chen, S. Y.P. | 2019 | Urban Nature Experiences Reduce Stress in the Context of Daily Life Based on Salivary Biomarkers | 10.3389/fpsyg.2019.00722 |
| Hystad, P.; Payette, Y.; Noisel, N.; Boileau, C. | 2019 | Green space associations with mental health and cognitive function: Results from the Quebec CARTaGENE cohort | 10.1097/EE9.0000000000000040 |
| Iqbal, A.; Mansell, W. | 2021 | A Thematic Analysis of Multiple Pathways Between Nature Engagement Activities and Well-Being | 10.3389/fpsyg.2021.580992 |
| Janeczko, E.; Bielinis, E.; Wojcik, R.; Woznicka, M.; Kedziora, W.; Lukowski, A.; Elsadek, M.; Szyc, K.; Janeczko, K. | 2020 | When Urban Environment Is Restorative: The Effect of Walking in Suburbs and Forests on Psychological and Physiological Relaxation of Young Polish Adults | 10.3390/f11050591 |
| Jang, S.; So, W.-Y. | 2017 | The effect of short-term outdoor taekwondo training on the concentration and mood of taekwondo players | 10.22374/1875-6859.13.2.9 |
| Jarvis, I.; Koehoorn, M.; Gergel, S. E.; van den Bosch, M. | 2020 | Different types of urban natural environments influence various dimensions of self-reported health | 10.1016/j.envres.2020.109614 |
| Jato-Espino, Daniel; Moscardó, Vanessa; Vallina Rodríguez, Alejandro; Lázaro, Esther | 2022 | Spatial statistical analysis of the relationship between self-reported mental health during the COVID-19 lockdown and closeness to green infrastructure | 10.1016/j.ufug.2021.127457 |
| Jiang, Xiangrong; Larsen, Linda; Sullivan, William | 2020 | Connections-between Daily Greenness Exposure and Health Outcomes | 10.3390/ijerph17113965 |
| Jimenez, M. P.; Elliott, E. G.; DeVille, N. V.; Laden, F.; Hart, J. E.; Weuve, J.; Grodstein, F.; James, P. | 2022 | Residential Green Space and Cognitive Function in a Large Cohort of Middle-Aged Women | 10.1001/jamanetworkopen.2022.9306 |
| Jin, X. R.; Shu, C.; Zeng, Y.; Liang, L. M.; Ji, J. S. | 2021 | Interaction of greenness and polygenic risk score of Alzheimer's disease on risk of cognitive impairment | 10.1016/j.scitotenv.2021.148767 |
| Jo, H. I.; Lee, K.; Jeon, J. Y. | 2022 | Effect of noise sensitivity on psychophysiological response through monoscopic 360 video and stereoscopic sound environment experience: a randomized control trial | 10.1038/s41598-022-08374-y |
| Jo, H.; Ikei, H.; Song, C.; Miyazaki, Y. | 2020 | Individual differences in the psychological effects of forest sounds based on type A and type B behavior patterns | 10.1016/j.ufug.2020.126855 |
| Jo, H.; Song, C. R.; Ikei, H.; Enomoto, S.; Kobayashi, H.; Miyazaki, Y. | 2019 | Physiological and Psychological Effects of Forest and Urban Sounds Using High-Resolution Sound Sources | 10.3390/ijerph16152649 |
| Johnson, Katherine A.; Pontvianne, Annabelle; Ly, Vi; Jin, Rui; Januar, Jonathan Haris; Machida, Keitaro; Sargent, Leisa D.; Lee, Kate E.; Williams, Nicholas S. G.; Williams, Kathryn J. H. | 2022 | Water and Meadow Views Both Afford Perceived but Not Performance-Based Attention Restoration: Results From Two Experimental Studies | 10.3389/fpsyg.2022.809629 |
| Jones, M. V.; Gidlow, C. J.; Hurst, G.; Masterson, D.; Smith, G.; Ellis, N.; Clark-Carter, D.; Tarvainen, M. P.; Braithwaite, E. C.; Nieuwenhuijsen, M. | 2021 | Psycho-physiological responses of repeated exposure to natural and urban environments | 10.1016/j.landurbplan.2021.104061 |
| Juanhong Yu; Kai Keng Ang; Su Hui Ho; Sia, A.; Ho, R. | 2017 | Prefrontal cortical activation while viewing urban and garden scenes: A pilot fNIRS study | 10.1109/EMBC.2017.8037376 |
| Kajosaari, A.; Pasanen, T. P. | 2021 | Restorative benefits of everyday green exercise: A spatial approach | 10.1016/j.landurbplan.2020.103978 |
| Kanelli, A. A.; Dimitrakopoulos, P. G.; Fyllas, N. M.; Chrousos, G. P.; Kalantzi, O. I. | 2021 | Engaging the Senses: The Association of Urban Green Space with General Health and Well-Being in Urban Residents | 10.3390/su13137322 |
| Kang, Y.; Kim, E. J. | 2019 | Differences of Restorative Effects While Viewing Urban Landscapes and Green Landscapes | 10.3390/su11072129 |
| Keijzer, C. de; Tonne, C.; Basagana, X.; Valentin, A.; Singh-Manoux, A.; Alonso, J.; Anto, J. M.; Nieuwenhuijsen, M. J.; Sunyer, J.; Dadvand, P. | 2018 | Residential Surrounding Greenness and Cognitive Decline: A 10-Year Follow-up of the Whitehall II Cohort | 10.1289/EHP2875 |
| Kim, J.; Park, D. B.; Seo, J. I. | 2020 | Exploring the Relationship between Forest Structure and Health | 10.3390/f11121264 |
| Kim, Jayeun; Kim, H. O. | 2017 | Demographic and Environmental Factors Associated with Mental Health: A Cross-Sectional Study | 10.3390/ijerph14040431 |
| Kingsley, J.; Foenander, E.; Bailey, A. | 2019 | "You feel like you're part of something bigger": exploring motivations for community garden participation in Melbourne, Australia | 10.1186/s12889-019-7108-3 |
| Klein, Y.; Lindfors, P.; Osika, W.; Hanson, L. L.M.; Stenfors, C. U.D. | 2022 | Residential Greenspace Is Associated with Lower Levels of Depressive and Burnout Symptoms, and Higher Levels of Life Satisfaction: A Nationwide Population-Based Study in Sweden | 10.3390/ijerph19095668 |
| Klompmaker, J. O.; Hoek, G.; Bloemsma, L. D.; Wijga, A. H.; van den Brink, C.; Brunekreef, B.; Lebret, E.; Gehring, U.; Janssen, N. A.H. | 2019 | Associations of combined exposures to surrounding green, air pollution and traffic noise on mental health | 10.1016/j.envint.2019.05.040 |
| Kobayashi, H.; Ikei, H.; Song, C.; Kagawa, T.; Miyazaki, Y. | 2021 | Comparing the impact of forest walking and forest viewing on psychological states | 10.1016/j.ufug.2020.126920 |
| Kondo, M. C.; Triguero-Mas, M.; Donaire-Gonzalezi, D.; Seto, E.; Valentin, A.; Hurst, G.; Carrasco-Turigas, G.; Masterson, D.; Ambros, A.; Ellis, N.; Swart, W.; Davis, N.; Maas, J.; Jerrett, M.; Gidlow, C. J.; Nieuwenhuijsen, M. J. | 2020 | Momentary mood response to natural outdoor environments in four European cities | 10.1016/j.envint.2019.105237 |
| Kristine, E.; Bocker, P. C.; Lars, A.; Constantinos, T.; Bo, M. P.; Jens-Christian, S. | 2018 | Childhood exposure to green space - A novel risk-decreasing mechanism for schizophrenia? | 10.1016/j.schres.2018.03.026 |
| Kuhn, S.; Duzel, S.; Eibich, P.; Krekel, C.; Wustemann, H.; Kolbe, J.; Martensson, J.; Goebel, J.; Gallinat, J.; Wagner, G. G.; Lindenberger, U. | 2017 | In search of features that constitute an "enriched environment" in humans: Associations between geographical properties and brain structure | 10.1038/s41598-017-12046-7 |
| Kuhn, S.; Duzel, S.; Mascherek, A.; Eibich, P.; Krekel, C.; Kolbe, J.; Goebel, J.; Gallinat, J.; Wagner, G. G.; Lindenberger, U. | 2021 | Urban green is more than the absence of city: Structural and functional neural basis of urbanicity and green space in the neighbourhood of older adults | 10.1016/j.lurbplan.2021.104196 |
| Kuhn, S.; Forlim, C. G.; Lender, A.; Wirtz, J.; Gallinat, J. | 2021 | Brain functional connectivity differs when viewing pictures from natural and built environments using fMRI resting state analysis | 10.1038/s41598-021-83246-5 |
| Kwok, S. W.H.; Wu, C. S.T.; Tong, H. T.; Ho, C. N.; Leung, K. L.; Leung, Y. C.P.; Lui, K. C.; Wong, C. K.C. | 2021 | Effects of the School-Based Integrated Health Promotion Program With Hydroponic Planting on Green Space Use and Satisfaction, Dietary Habits, and Mental Health in Early Adolescent Students: A Feasibility Quasi-Experiment | 10.3389/fpubh.2021.740102 |
| Lee, D. G.; Lee, M. M.; Jeong, Y. M.; Kim, J. G.; Yoon, Y. K.; Shin, W. S. | 2021 | Influence of Forest Visitors' Perceived Restorativeness on Social-Psychological Stress | 10.3390/ijerph18126328 |
| Lee, E.; Bailey, A. W.; Hungenberg, E.; Demastus, C.; Kang, H. K. | 2021 | Comparison of psychological states of runners within urban and nature environments: the application of electroencephalography (EEG), geographic information systems (GIS), and fitness tracker | 10.1080/03623319.2021.1960044 |
| Lee, H. J.; Lee, D. K. | 2019 | Do Sociodemographic Factors and Urban Green Space Affect Mental Health Outcomes Among the Urban Elderly Population? | 10.3390/ijerph16050789 |
| Lee, J. | 2017 | Experimental study on the health benefits of garden landscape | 10.3390/ijerph14070829 |
| Li, D. Y.; Deal, B.; Zhou, X. L.; Slavenas, M.; Sullivan, W. C. | 2018 | Moving beyond the neighborhood: Daily exposure to nature and adolescents' mood | 10.1016/j.landurbplan.2018.01.009 |
| Li, D. Y.; Zhai, Y. J.; Xiao, Y. Y.; Newman, G.; Wang, D. | 2019 | Subtypes of park use and self-reported psychological benefits among older adults: A multilevel latent class analysis approach | 10.1016/j.landurbplan.2019.103605 |
| Li, H. Q.D.; Dong, W. Y.; Wang, Z. M.; Chen, N.; Wu, J. P.; Wang, G. X.; Jiang, T. | 2021 | Effect of a Virtual Reality-Based Restorative Environment on the Emotional and Cognitive Recovery of Individuals with Mild-to-Moderate Anxiety and Depression | 10.3390/ijerph18179053 |
| Li, H. S.; Liu, H. W.; Yang, Z. Q.; Bi, S. L.; Cao, Y.; Zhang, G. D. | 2021 | The Effects of Green and Urban Walking in Different Time Frames on Physio-Psychological Responses of Middle-Aged and Older People in Chengdu, China | 10.3390/ijerph18010090 |
| Li, H.; Xie, H.; Woodward, G. | 2021 | Soundscape components, perceptions, and EEG reactions in typical mountainous urban parks | 10.1016/j.ufug.2021.127269 |
| Li, Z. M.; Zhang, W. Z.; Wang, L. S.; Liu, H. | 2022 | Regulating effects of the biophilic environment with strawberry plants on psychophysiological health and cognitive performance in small spaces | 10.1016/j.buildenv.2022.108801 |
| Lin, W.; Chen, Q. B.; Jiang, M. Y.; Tao, J. Y.; Liu, Z. F.; Zhang, X. X.; Wu, L. J.; Xu, S.; Kang, Y. S.; Zeng, Q. Y. | 2020 | Sitting or Walking? Analyzing the Neural Emotional Indicators of Urban Green Space Behavior with Mobile EEG | 10.1007/s11524-019-00407-8 |
| Lin, W.; Chen, Q. B.; Jiang, M. Y.; Zhang, X. X.; Liu, Z. F.; Tao, J. Y.; Wu, L. J.; Xu, S.; Kang, Y. S.; Zeng, Q. Y. | 2019 | The effect of green space behaviour and per capita area in small urban green spaces on psychophysiological responses | 10.1016/j.landurbplan.2019.103637 |
| Liu, H. X.; Li, F.; Li, J. Y.; Zhang, Y. Y. | 2017 | The relationships between urban parks, residents' physical activity, and mental health benefits: A case study from Beijing, China | 10.1016/j.jenvman.2016.12.058 |
| Liu, H. X.; Ren, H.; Remme, R. P.; Nong, H. F.; Sui, C. H. | 2021 | The effect of urban nature exposure on mental health-a case study of Guangzhou | 10.1016/j.jclepro.2021.127100 |
| Liu, L. H.; Qu, H. Y.; Ma, Y. M.; Wang, K.; Qu, H. X. | 2022 | Restorative benefits of urban green space: Physiological, psychological restoration and eye movement analysis | 10.1016/j.jenvman.2021.113930 |
| Liu, P.; Liu, M. N.; Xia, T. T.; Wang, Y. T.; Guo, P. | 2021 | The Relationship between Landscape Metrics and Facial Expressions in 18 Urban Forest Parks of Northern China | 10.3390/f12121619 |
| Liu, P.; Liu, M. N.; Xia, T. T.; Wang, Y. T.; Wei, H. X. | 2021 | Can Urban Forest Settings Evoke Positive Emotion? Evidence on Facial Expressions and Detection of Driving Factors | 10.3390/su13168687 |
| Liu, Q. H.; Wang, X. P.; Liu, J. L.; An, C. Y.; Liu, Y. Q.; Fan, X. L.; Hu, Y. S. | 2021 | Physiological and Psychological Effects of Nature Experiences in Different Forests on Young People | 10.3390/f12101391 |
| Liu, Q. H.; Wang, X. P.; Liu, J. L.; Zhang, G. L.; An, C. Y.; Liu, Y. Q.; Fan, X. L.; Hu, Y. S.; Zhang, H. | 2021 | The Relationship between the Restorative Perception of the Environment and the Physiological and Psychological Effects of Different Types of Forests on University Students | 10.3390/ijerph182212224 |
| Liu, Y.; Wang, R. Y.; Xiao, Y.; Huang, B. S.; Chen, H. S.; Li, Z. G. | 2019 | Exploring the linkage between greenness exposure and depression among Chinese people: Mediating roles of physical activity, stress and social cohesion and moderating role of urbanicity | 10.1016/j.healthplace.2019.102168 |
| Lopes, S.; Lima, M.; Silva, K. | 2020 | Nature can get it out of your mind: The rumination reducing effects of contact with nature and the mediating role of awe and mood | 10.1016/j.jenvp.2020.101489 |
| Lotfi, Y. A.; Refaat, M.; El Attar, M.; Salam, A. A. | 2020 | Vertical gardens as a restorative tool in urban spaces of New Cairo | 10.1016/j.asej.2019.12.004 |
| Lu, J. C.; Luo, X. L.; Yang, N. N.; Shen, Y. | 2021 | Multiple Pathways: The Influence Mechanism of Greenspace Exposure on Mental Health-A Case Study of Hangzhou, China | 10.3390/land10040339 |
| Maes, M. J.A.; Pirani, M.; Booth, E. R.; Shen, C.; Milligan, B.; Jones, K. E.; Toledano, M. B. | 2021 | Benefit of woodland and other natural environments for adolescents' cognition and mental health | 10.1038/s41893-021-00751-1 |
| Mancus, G.; Cimino, an; Hasan, M. Z.; Campbell, J. C.; Winch, P. J.; Sharps, P.; Tsuyuki, K.; Da Granger; Stockman, J. K. | 2021 | Residential Greenness Positively Associated with the Cortisol to DHEA Ratio among Urban-Dwelling African American Women at Risk for HIV | 10.1007/s11524-020-00492-0 |
| Marois, A.; Charbonneau, B.; Szolosi, am; Watson, J. M. | 2021 | The Differential Impact of Mystery in Nature on Attention: An Oculometric Study | 10.3389/fpsyg.2021.759616 |
| Marques, P.; Silva, A. S.; Quaresma, Y.; Manna, L. R.; Neto, N. D.; Mazzoni, R. | 2021 | Home gardens can be more important than other urban green infrastructure for mental well-being during COVID-19 pandemics | 10.1016/j.ufug.2021.127268 |
| Marselle; Bowler, de; Watzema, J.; Eichenberg, D.; Kirsten, T.; Bonn, A. | 2020 | Urban street tree biodiversity and antidepressant prescriptions | 10.1038/s41598-020-79924-5 |
| Martin, L.; Pahl, S.; White, M. P.; May, J. | 2019 | Natural environments and craving: The mediating role of negative affect | 10.1016/j.healthplace.2019.102160 |
| Maury-Mora, M.; Gomez-Villarino, M. T.; Varela-Martinez, C. | 2022 | Urban green spaces and stress during COVID-19 lockdown: A case study for the city of Madrid | 10.1016/j.ufug.2022.127492 |
| Mavoa, S.; Lucassen, M.; Denny, S.; Utter, J.; Clark, T.; Smith, M. | 2019 | Natural neighbourhood environments and the emotional health of urban New Zealand adolescents | 10.1016/j.landurbplan.2019.103638 |
| McAllister, E.; Bhullar, N.; Schutte, N. S. | 2017 | Into the Woods or a Stroll in the Park: How Virtual Contact with Nature Impacts Positive and Negative Affect | 10.3390/ijerph14070786 |
| Mears, M.; Brindley, P.; Jorgensen, A.; Maheswaran, R. | 2020 | Population-level linkages between urban greenspace and health inequality: The case for using multiple indicators of neighbourhood greenspace | 10.1016/j.healthplace.2020.102284 |
| Meidenbauer, Kimberly L.; Stenfors, Cecilia U. D.; Bratman, Gregory N.; Gross, James J.; Schertz, Kathryn E.; Choe, Kyoung Whan; Berman, Marc G. | 2020 | The affective benefits of nature exposure: What's nature got to do with it? | 10.1016/j.jenvp.2020.101498 |
| Memari, S.; Pazhouhanfar, M.; Grahn, P. | 2021 | Perceived Sensory Dimensions of Green Areas: An Experimental Study on Stress Recovery | 10.3390/su13105419 |
| Meng, Q.; Hu, X.; Kang, J.; Wu, Y. | 2020 | On the effectiveness of facial expression recognition for evaluation of urban sound perception | 10.1016/j.scitotenv.2019.135484 |
| Mennis, J.; Li, X. J.; Meenar, M.; Coatsworth, J. D.; McKeon, T. P.; Mason, M. J. | 2021 | Residential Greenspace and Urban Adolescent Substance Use: Exploring Interactive Effects with Peer Network Health, Sex, and Executive Function | 10.3390/ijerph18041611 |
| Mennis, J.; Mason, M.; Ambrus, A. | 2018 | Urban greenspace is associated with reduced psychological stress among adolescents: A Geographic Ecological Momentary Assessment (GEMA) analysis of activity space | 10.1016/j.landurbplan.2018.02.008 |
| Menzel, C.; Dennenmoser, F.; Reese, G. | 2020 | Feeling Stressed and Ugly? Leave the City and Visit Nature! An Experiment on Self- and Other-Perceived Stress and Attractiveness Levels | 10.3390/ijerph17228519 |
| Methorst, J.; Bonn, A.; Marselle, M.; Bohning-Gaese, K.; Rehdanz, K. | 2021 | Species richness is positively related to mental health - A study for Germany | 10.1016/j.landurbplan.2021.104084 |
| Meyer-GrandBastien, A.; Burel, F.; Hellier, E.; Bergerot, B. | 2020 | A step towards understanding the relationship between species diversity and psychological restoration of visitors in urban green spaces using landscape heterogeneity | 10.1016/j.landurbplan.2019.103728 |
| Michels, N.; Witte, F. de; Di Bisceglie, E.; Seynhaeve, M.; Vandebuerie, T. | 2021 | Green nature effect on stress response and stress eating in the lab: Color versus environmental content | 10.1016/j.envres.2020.110589 |
| Min, K. B.; Kim, H. J.; Min, J. Y. | 2017 | Parks and green areas and the risk for depression and suicidal indicators | 10.1007/s00038-017-0958-5 |
| Mintz, K. K.; Ayalon, O.; Nathan, O.; Eshet, T. | 2021 | See or Be? Contact with nature and well-being during COVID-19 lockdown | 10.1016/j.jenvp.2021.101714 |
| Mokhtar, D.; Aziz, N. A. A.; Mariapan, M. | 2018 | Physiological and Psychological Health Benefits of Urban Green Space in Kuala Lumpur: A comparison between Taman Botani Perdana and Jalan Bukit Bintang |  |
| Moreira, T. C.L.; Polize, J. L.; Brito, M.; Da Silva, D. F.; Chiavegato, A. D. P.; Viana, M. C.; Andrade, L. H.; Mauad, T. | 2022 | Assessing the impact of urban environment and green infrastructure on mental health: results from the Sao Paulo Megacity Mental Health Survey | 10.1038/s41370-021-00349-x |
| Mostajeran, F.; Krzikawski, J.; Steinicke, F.; Kuhn, S. | 2021 | Effects of exposure to immersive videos and photo slideshows of forest and urban environments | 10.1038/s41598-021-83277-y |
| Moyle, W.; Jones, C.; Dwan, T.; Petrovich, T. | 2018 | Effectiveness of a Virtual Reality Forest on People With Dementia: A Mixed Methods Pilot Study | 10.1093/geront/gnw270 |
| Mueller, M. A.E.; Flouri, E.; Kokosi, T. | 2019 | The role of the physical environment in adolescent mental health | 10.1016/j.healthplace.2019.102153 |
| Mukherjee, D.; Safraj, S.; Tayyab, M.; Shivashankar, R.; Patel, S. A.; Narayanan, G.; Ajay, V. S.; Ali, M. K.; Narayan, K. M.V.; Tandon, N.; Prabhakaran, D. | 2017 | Park availability and major depression in individuals with chronic conditions: Is there an association in urban India? | 10.1016/j.healthplace.2017.07.004 |
| Murphy, L. F.; Kalpakjian, C.; Charlifue, S.; Heinemann, A. W.; Slavin, M.; Rohrbach, T.; Tulsky, D. S.; Botticello, A. L. | 2022 | Greener on the other side? an analysis of the association between residential greenspace and psychological well-being among people living with spinal cord injury in the United States | 10.1038/s41393-021-00736-z |
| Nath, T. K.; Han, S. S.Z.; Lechner, am | 2018 | Urban green space and well-being in Kuala Lumpur, Malaysia | 10.1016/j.ufug.2018.09.013 |
| Nawrath, M.; Elsey, H.; Dallimer, M. | 2022 | Why cultural ecosystem services matter most: Exploring the pathways linking greenspaces and mental health in a low-income country | 10.1016/j.scitotenv.2021.150551 |
| Nazif-Munoz, J. I.; Laurent, J. G. C.; Browning, M.; Spengler, J.; Olvera Álvarez, H. A. | 2020 | Green, brown, and gray: Associations between different measurements of land patterns and depression among nursing students in El Paso, Texas | 10.3390/ijerph17218146 |
| Neale, C.; Lopez, S.; Roe, J. | 2021 | Psychological Restoration and the Effect of People in Nature and Urban Scenes: A Laboratory Experiment | 10.3390/su13116464 |
| Neilson, B. N.; Craig, C. M.; Curiel, R. Y.; Klein, M. I. | 2021 | Restoring Attentional Resources With Nature: A Replication Study of Berto's (2005) Paradigm Including Commentary From Dr. Rita Berto | 10.1177/0018720820909287 |
| Nghiem, T. P.L.; Wong, K. L.; Jeevanandam, L.; Chang, C. C.; Tan, L. Y.C.; Goh, Y.; Carrasco, L. R. | 2021 | Biodiverse urban forests, happy people: Experimental evidence linking perceived biodiversity, restoration, and emotional wellbeing | 10.1016/j.ufug.2021.127030 |
| Nichani, V.; Dirks, K.; Burns, B.; Bird, A.; Grant, C. | 2017 | Green space and depression during pregnancy: Results from the growing up in New Zealand study | 10.3390/ijerph14091083 |
| Nishigaki, M.; Hanazato, M.; Koga, C.; Kondo, K. | 2020 | What types of greenspaces are associated with depression in urban and rural older adults?: A multilevel cross-sectional study from JAGES | 10.3390/ijerph17249276 |
| Noel, C.; Rodriguez-Loureiro, L.; Vanroelen, C.; Gadeyne, S. | 2021 | Perceived Health Impact and Usage of Public Green Spaces in Brussels' Metropolitan Area During the COVID-19 Epidemic | 10.3389/frsc.2021.668443 |
| Noordzij, J. M.; Beenackers, M. A.; Groeniger, J. O.; Timmermans, E.; Chaix, B.; Doiron, D.; Huisman, M.; Motoc, I.; Ruiz, M.; Wissa, R.; Avendano, M.; van Lenthe, F. J. | 2021 | Green spaces, subjective health and depressed affect in middle-aged and older adults: a cross-country comparison of four European cohorts | 10.1136/jech-2020-214257 |
| Noordzij, J. M.; Beenackers, M. A.; Groeniger, J. O.; van Lenthe, F. J. | 2020 | Effect of changes in green spaces on mental health in older adults: a fixed effects analysis | 10.1136/jech-2019-212704 |
| Nordh, H.; Evensen, K. H.; Skar, M. | 2017 | A peaceful place in the city-A qualitative study of restorative components of the cemetery | 10.1016/j.landurbplan.2017.06.004 |
| Ochiai, H.; Song, C. R.; Jo, H.; Oishi, M.; Imai, M.; Miyazaki, Y. | 2020 | Relaxing Effect Induced by Forest Sound in Patients with Gambling Disorder | 10.3390/su12155969 |
| Oh, R. R.Y.; Fielding, K. S.S.; Chang, C. C.; Nghiem, L. P.; Tan, C. L.Y.; Quazi, S. A.; Shanahan, D. F.; Gaston, K. J.; Carrasco, R. L.; Fuller, R. A. | 2021 | Health and Wellbeing Benefits from Nature Experiences in Tropical Settings Depend on Strength of Connection to Nature | 10.3390/ijerph181910149 |
| Ojala, A.; Korpela, K.; Tyrvainen, L.; Tiittanen, P.; Lanki, T. | 2019 | Restorative effects of urban green environments and the role of urban-nature orientedness and noise sensitivity: A field experiment | 10.1016/j.healthplace.2018.11.004 |
| Olszewska-Guizzo, A.; Escoffier, N.; Chan, J.; Yok, T. P. | 2018 | Window view and the brain: Effects of floor level and green cover on the alpha and beta rhythms in a passive exposure eeg experiment | 10.3390/ijerph15112358 |
| Olszewska-Guizzo, A.; Fogel, A.; Escoffier, N.; Ho, R. | 2021 | Effects of COVID-19-related stay-at-home order on neuropsychophysiological response to urban spaces: Beneficial role of exposure to nature? | 10.1016/j.jenvp.2021.101590 |
| Olszewska-Guizzo, A.; Fogel, A.; Escoffier, N.; Sia, A.; Nakazawa, K.; Kumagai, A.; Dan, I. P.T.; Ho, R. G. | 2022 | Therapeutic Garden With Contemplative Features Induces Desirable Changes in Mood and Brain Activity in Depressed Adults | 10.3389/fpsyt.2022.757056 |
| Olszewska-Guizzo, A.; Sia, A.; Fogel, A.; Ho, R. | 2020 | Can Exposure to Certain Urban Green Spaces Trigger Frontal Alpha Asymmetry in the Brain?-Preliminary Findings from a Passive Task EEG Study | 10.3390/ijerph17020394 |
| Orstad, S. L.; Szuhany, K.; Tamura, K.; Le Thorpe; Jay, M. | 2020 | Park Proximity and Use for Physical Activity among Urban Residents: Associations with Mental Health | 10.3390/ijerph17134885 |
| Osborne, T. |  | Restorative and Afflicting Qualities of the Microspace Encounter: Psychophysiological Reactions to the Spaces of the City | 10.1080/24694452.2021.1972791 |
| Park, S. H.; Petrunoff, N. A.; Wang, N. X.; van Dam, R. M.; Sia, A.; Tan, C. S.; Muller-Riemenschneider, F. | 2022 | Daily park use, physical activity, and psychological stress: A study using smartphone-based ecological momentary assessment amongst a multi-ethnic Asian cohort | 10.1016/j.mhpa.2022.100440 |
| Paul, L. A.; Hystad, P.; Burnett, R. T.; Kwong, J. C.; Crouse, D. L.; van Donkelaar, A.; Tu, K.; Lavigne, E.; Copes, R.; Martin, R. V.; Chen, H. | 2020 | Urban green space and the risks of dementia and stroke | 10.1016/j.envres.2020.109520 |
| Pelgrims, Ingrid; Devleesschauwer, Brecht; Guyot, Madeleine; Keune, Hans; Nawrot, Tim S.; Remmen, Roy; Saenen, Nelly D.; Trabelsi, Sonia; Thomas, Isabelle; Aerts, Raf; Clercq, Eva M. de | 2021 | Association between urban environment and mental health in Brussels, Belgium | 10.1186/s12889-021-10557-7 |
| Peng, W. J.; Shi, H. Y.; Li, M. Y.; Li, X. H.; Liu, T.; Wang, Y. | 2022 | Association of residential greenness with geriatric depression among the elderly covered by long-term care insurance in Shanghai | 10.1007/s11356-021-16585-5 |
| Pfeiffer, Jack A.; Hart, Joy L.; Wood, Lindsey A.; Bhatnagar, Aruni; Keith, Rachel J.; Yeager, Ray A.; Smith, Ted; Tomlinson, Madeline; Gilkey, Delana; Kerstiens, Savanna; Gao, Hong; Srivastava, Sanjay; Walker, Kandi L. | 2021 | The importance of urban planning: Views of greenness and open space is reversely associated with self-reported views and depressive symptoms | 10.18332/popmed/139173 |
| Pope, D.; Tisdall, R.; Middleton, J.; Verma, A.; van Ameijden, E.; Birt, C.; Macherianakis, A.; Bruce, N. G. | 2018 | Quality of and access to green space in relation to psychological distress: results from a population-based cross-sectional study as part of the EURO-URHIS 2 project | 10.1093/eurpub/ckv094 |
| Pratiwi, P. I.; Xiang, Q. Y.; Furuya, K. | 2019 | Physiological and Psychological Effects of Viewing Urban Parks in Different Seasons in Adults | 10.3390/ijerph16214279 |
| Pratiwi, P. I.; Xiang, Q. Y.; Furuya, K. | 2020 | Physiological and Psychological Effects of Walking in Urban Parks and Its Imagery in Different Seasons in Middle-Aged and Older Adults: Evidence from Matsudo City, Japan | 10.3390/su12104003 |
| Puhakka, R. | 2021 | University students' participation in outdoor recreation and the perceived well-being effects of nature | 10.1016/j.jort.2021.100425 |
| Qiao, Y. H.; Chen, Z. N.; Chen, Y. Q.; Zheng, T. X. | 2021 | Deciphering the Link Between Mental Health and Green Space in Shenzhen, China: The Mediating Impact of Residents' Satisfaction | 10.3389/fpubh.2021.561809 |
| Qin, B.; Zhu, W.; Wang, J. J.; Peng, Y. Y. | 2021 | Understanding the relationship between neighbourhood green space and mental wellbeing: A case study of Beijing, China | 10.1016/j.cities.2020.103039 |
| Qiu, L.; Chen, Q. J.; Gao, T. | 2021 | The Effects of Urban Natural Environments on Preference and Self-Reported Psychological Restoration of the Elderly | 10.3390/ijerph18020509 |
| Raman, T. L.; Aziz, N. A.A.; Yaakob, S. S.N. | 2021 | The Effects of Different Natural Environment Influences on Health and Psychological Well-Being of People: A Case Study in Selangor | 10.3390/su13158597 |
| Rantakokko, M.; Keskinen, K. E.; Kokko, K.; Portegijs, E. | 2018 | Nature diversity and well-being in old age | 10.1007/s40520-017-0797-5 |
| Reese, G.; Stahlberg, J.; Menzel, C. |  | Digital shinrin-yoku: do nature experiences in virtual reality reduce stress and increase well-being as strongly as similar experiences in a physical forest? | 10.1007/s10055-022-00631-9 |
| Reuben, A.; Arseneault, L.; Belsky, D. W.; Caspi, A.; Fisher, H. L.; Houts, R. M.; Moffitt, T. E.; Odgers, C. | 2019 | Residential neighborhood greenery and children's cognitive development | 10.1016/j.socscimed.2019.04.029 |
| Ribeiro, A. I.; Triguero-Mas, M.; Santos, C. J.; Gomez-Nieto, A.; Cole, H.; Anguelovski, I.; Silva, F. M.; Baro, F. | 2021 | Exposure to nature and mental health outcomes during COVID-19 lockdown. A comparison between Portugal and Spain | 10.1016/j.envint.2021.106664 |
| Roberts, H.; Helbich, M. | 2021 | Multiple environmental exposures along daily mobility paths and depressive symptoms: A smartphone-based tracking study | 10.1016/j.envint.2021.106635 |
| Roberts, M.; Irvine, K. N.; McVittie, A. | 2021 | Associations between greenspace and mental health prescription rates in urban areas | 10.1016/j.ufug.2021.127301 |
| Roe, J. J.; Aspinall, P. A.; Thompson, C. W. | 2017 | Coping with Stress in Deprived Urban Neighborhoods: What Is the Role of Green Space According to Life Stage? | 10.3389/fpsyg.2017.01760 |
| Roe, J.; Mondschein, A.; Neale, C.; Barnes, L.; Boukhechba, M.; Lopez, S. | 2020 | The Urban Built Environment, Walking and Mental Health Outcomes Among Older Adults: A Pilot Study | 10.3389/fpubh.2020.575946 |
| Rugel, E. J.; Carpiano, R. M.; Henderson, S. B.; Brauer, M. | 2019 | Exposure to natural space, sense of community belonging, and adverse mental health outcomes across an urban region | 10.1016/j.envres.2019.01.034 |
| Ruijsbroek, A.; Droomers, M.; Kruize, H.; van Kempen, E.; Gidlow, C. J.; Hurst, G.; Andrusaityte, S.; Nieuwenhuijsen, M. J.; Maas, J.; Hardyns, W.; Stronks, K.; Groenewegen, P. P. | 2017 | Does the health impact of exposure to neighbourhood green space differ between population groups? An explorative study in four European cities | 10.3390/ijerph14060618 |
| Ruijsbroek, A.; Mohnen, S. M.; Droomers, M.; Kruize, H.; Gidlow, C.; Gražulevičiene, R.; Andrusaityte, S.; Maas, J.; Nieuwenhuijsen, M. J.; Triguero-Mas, M.; Masterson, D.; Ellis, N.; van Kempen, E.; Hardyns, W.; Stronks, K.; Groenewegen, P. P. | 2017 | Neighbourhood green space, social environment and mental health: an examination in four European cities | 10.1007/s00038-017-0963-8 |
| Samus, A.; Freeman, C.; Dickinson, K. J.M.; van Heezik, Y. | 2022 | Relationships between nature connectedness, biodiversity of private gardens, and mental well-being during the Covid-19 lockdown | 10.1016/j.ufug.2022.127519 |
| Sarkar, C.; Webster, C.; Gallacher, J. | 2018 | Residential greenness and prevalence of major depressive disorders: a cross-sectional, visualisational, associational study of 94 879 adult UK Biobank participants | 10.1016/S2542-5196(18)30051-2 |
| Schebella, M. F.; Weber, D.; Schultz, L.; Weinstein, P. | 2020 | The Nature of Reality: Human Stress Recovery during Exposure to Biodiverse, Multisensory Virtual Environments | 10.3390/ijerph17010056 |
| Schutte, N. S.; Bhullar, N.; Stilinovic, E. J.; Richardson, K. | 2017 | The Impact of Virtual Environments on Restorativeness and Affect | 10.1089/eco.2016.0042 |
| Sefcik, J. S.; Kondo, M. C.; Klusaritz, H.; Sarantschin, E.; Solomon, S.; Roepke, A.; South, E. C.; Jacoby, S. F. | 2019 | Perceptions of Nature and Access to Green Space in Four Urban Neighborhoods | 10.3390/ijerph16132313 |
| Shrestha, T.; Di Blasi, Z.; Cassarino, M. | 2021 | Natural or Urban Campus Walks and Vitality in University Students: Exploratory Qualitative Findings from a Pilot Randomised Controlled Study | 10.3390/ijerph18042003 |
| Slawsky, E. D.; Hajat, A.; Rhew, I. C.; Russette, H.; Semmens, E. O.; Kaufman, J. D.; Leary, C. S.; Fitzpatrick, A. L. | 2022 | Neighborhood greenspace exposure as a protective factor in dementia risk among U.S. adults 75 years or older: a cohort study | 10.1186/s12940-022-00830-6 |
| Soga, M.; Cox, D. T.C.; Yamaura, Y.; Gaston, K. J.; Kurisu, K.; Hanaki, K. | 2017 | Health Benefits of Urban Allotment Gardening: Improved Physical and Psychological Well-Being and Social Integration | 10.3390/ijerph14010071 |
| Soga, M.; Evans, M. J.; Tsuchiya, K.; Fukano, Y. | 2021 | A room with a green view: the importance of nearby nature for mental health during the COVID-19 pandemic | 10.1002/eap.2248 |
| Song, C. R.; Ikei, H.; Kagawa, T.; Miyazaki, Y. | 2019 | Physiological and Psychological Effects of Viewing Forests on Young Women | 10.3390/f10080635 |
| Song, C. R.; Ikei, H.; Kagawa, T.; Miyazaki, Y. | 2020 | Effect of Viewing Real Forest Landscapes on Brain Activity | 10.3390/su12166601 |
| Song, C. R.; Ikei, H.; Park, B. J.; Lee, J.; Kagawa, T.; Miyazaki, Y. | 2018 | Psychological Benefits of Walking through Forest Areas | 10.3390/ijerph15122804 |
| Song, C.; Ikei, H.; Kagawa, T.; Miyazaki, Y. | 2019 | Effects of Walking in a Forest on Young Women | 10.3390/ijerph16020229 |
| Song, C.; Ikei, H.; Park, B. J.; Lee, J.; Kagawa, T.; Miyazaki, Y. | 2020 | Association between the Psychological Effects of Viewing Forest Landscapes and Trait Anxiety Level | 10.3390/ijerph17155479 |
| Song, H.; Lane, K. J.; Kim, H.; Byun, G.; Le, M.; Choi, Y.; Park, C. R.; Lee, J. T. | 2019 | Association between Urban Greenness and Depressive Symptoms: Evaluation of Greenness Using Various Indicators | 10.3390/ijerph16020173 |
| Souter-Brown, G.; Hinckson, E.; Duncan, S. | 2021 | Effects of a sensory garden on workplace wellbeing: A randomised control trial | 10.1016/j.landurbplan.2020.103997 |
| Stas, M.; Aerts, R.; Hendrickx, M.; Dendoncker, N.; Dujardin, S.; Linard, C.; Nawrot, T. S.; van Nieuwenhuyse, A.; Aerts, J. M.; van Orshoven, J.; Somers, B. | 2021 | Residential green space types, allergy symptoms and mental health in a cohort of tree pollen allergy patients | 10.1016/j.lurbplan.2021.104070 |
| Stewart, M.; Haaga, D. A.F. | 2018 | State Mindfulness as a Mediator of the Effects of Exposure to Nature on Affect and Psychological Well-Being | 10.1089/eco.2017.0033 |
| Stigsdotter, U. K.; Corazon, S. S.; Sidenius, U.; Kristiansen, J.; Grahn, P. | 2017 | It is not all bad for the grey city - A crossover study on physiological and psychological restoration in a forest and an urban environment | 10.1016/j.healthplace.2017.05.007 |
| Swierad, E. M.; Huang, T. T.K. | 2018 | An Exploration of Psychosocial Pathways of Parks' Effects on Health: A Qualitative Study | 10.3390/ijerph15081693 |
| Takayama, N.; Morikawa, T.; Bielinis, E. | 2019 | Relation between Psychological Restorativeness and Lifestyle, Quality of Life, Resilience, and Stress-Coping in Forest Settings | 10.3390/ijerph16081456 |
| Takayama, Norimasa; Fujiwara, Akio; Saito, Haruo; Horiuchi, Masahiro | 2017 | Management Effectiveness of a Secondary Coniferous Forest for Landscape Appreciation and Psychological Restoration | 10.3390/ijerph14070800 |
| Taylor, L.; Hahs, A. K.; Hochuli, D. F. | 2018 | Wellbeing and urban living: nurtured by nature | 10.1007/s11252-017-0702-1 |
| Theodorou, A.; Panno, A.; Carrus, G.; Carbone, G. A.; Massullo, C.; Imperatori, C. | 2021 | Stay home, stay safe, stay green: The role of gardening activities on mental health during the Covid-19 home confinement | 10.1016/j.ufug.2021.127091 |
| Thompson, A.; Bruk-Lee, V. | 2019 | Naturally! Examining Nature's Role in Workplace Strain Reduction | 10.1007/s41542-019-00033-5 |
| Thygesen, M.; Engemann, K.; Holst, G. J.; Hansen, B.; Geels, C.; Brandt, J.; Pedersen, C. B.; Dalsgaard, S. | 2020 | The Association between Residential Green Space in Childhood and Development of Attention Deficit Hyperactivity Disorder: A Population-Based Cohort Study | 10.1289/EHP6729 |
| Tiako, M. J.N.; South, E.; Shannon, M. M.; McCarthy, C.; Meisel, Z. F.; Elovitz, M. A.; Burris, H. H. | 2021 | Urban residential tree canopy and perceived stress among pregnant women | 10.1016/j.envres.2021.111620 |
| Toda, M. T.; Riol, A. A.; Cirach, M.; Estarlich, M.; Fernandez-Somoano, A.; Gonzalez-Safont, L.; Guxens, M.; Julvez, J.; Riano-Galan, I.; Sunyer, J.; Dadvand, P. | 2020 | Residential Surrounding Greenspace and Mental Health in Three Spanish Areas | 10.3390/ijerph17165670 |
| Tomao, A.; Secondi, L.; Carrus, G.; Corona, P.; Portoghesi, L.; Agrimi, M. | 2018 | Restorative urban forests: Exploring the relationships between forest stand structure, perceived restorativeness and benefits gained by visitors to coastal *Pinus pinea* forests | 10.1016/j.ecolind.2018.03.051 |
| Tomita, A.; Vandormael, am; Cuadros, D.; Di Minin, E.; Heikinheimo, V.; Tanser, F.; Slotow, R.; Burns, J. K. | 2017 | Green environment and incident depression in South Africa: a geospatial analysis and mental health implications in a resource-limited setting | 10.1016/S2542-5196(17)30063-3 |
| Tost, H.; Reichert, M.; Braun, U.; Reinhard, I.; Peters, R.; Lautenbach, S.; Hoell, A.; Schwarz, E.; Ebner-Priemer, U.; Zipf, A.; Meyer-Lindenberg, A. | 2019 | Neural correlates of individual differences in affective benefit of real-life urban green space exposure | 10.1038/s41593-019-0451-y |
| Triguero-Mas, M.; Anguelovski, I.; Cirac-Claveras, J.; Connolly, J.; Vazquez, A.; Urgell-Plaza, F.; Cardona-Giralt, N.; Sanye-Mengual, E.; Alonso, J.; Cole, H. | 2020 | Quality of Life Benefits of Urban Rooftop Gardening for People With Intellectual Disabilities or Mental Health Disorders | 10.5888/pcd17.200087 |
| Triguero-Mas, M.; Gidlow, C. J.; Martinez, D.; Bont, J. de; Carrasco-Turigas, G.; Martinez-Iniguez, T.; Hurst, G.; Masterson, D.; Donaire-Gonzalez, D.; Seto, E.; Jones, M. V.; Nieuwenhuijsen, M. J. | 2017 | The effect of randomised exposure to different types of natural outdoor environments compared to exposure to an urban environment on people with indications of psychological distress in Catalonia | 10.1371/journal.pone.0172200 |
| Triguero-Mas, Margarita; Donaire-Gonzalez, David; Seto, Edmund; Valentín, Antònia; Martínez, David; Smith, Graham; Hurst, Gemma; Carrasco-Turigas, Glòria; Masterson, Daniel; van den Berg, Magdalena; Ambròs, Albert; Martínez-Íñiguez, Tania; Dedele, Audrius; Ellis, Naomi; Grazulevicius, Tomas; Voorsmit, Martin; Cirach, Marta; Cirac-Claveras, Judith; Swart, Wim; Clasquin, Eddy; Ruijsbroek, Annemarie; Maas, Jolanda; Jerret, Michael; Gražulevičienė, Regina; Kruize, Hanneke; Gidlow, Christopher J.; Nieuwenhuijsen, Mark J. | 2017 | Natural outdoor environments and mental health: Stress as a possible mechanism | 10.1016/j.envres.2017.08.048 |
| Tsai, W. L.; McHale; Jennings, V.; Marquet, O.; Hipp, J. A.; Leung, Y. F.; Floyd, M. F. | 2018 | Relationships between Characteristics of Urban Green Land Cover and Mental Health in US Metropolitan Areas | 10.3390/ijerph15020340 |
| Uebel, K.; Marselle, M.; Dean, A. J.; Rhodes, [JR]; Bonn, A. | 2021 | Urban green space soundscapes and their perceived restorativeness | 10.1002/pan3.10215 |
| van den Berg, M.; van Poppel, M.; Smith, G.; Triguero-Mas, M.; Andrusaityte, S.; van Kamp, I.; van Mechelen, W.; Gidlow, C.; Grazuleviciene, R.; Nieuwenhuijsen, M. J.; Kruize, H.; Maas, J. | 2017 | Does time spent on visits to green space mediate the associations between the level of residential greenness and mental health? | 10.1016/j.ufug.2017.04.010 |
| van Hedger, S. C.; Nusbaum, H. C.; Clohisy, L.; Jaeggi, S. M.; Buschkuehl, M.; Berman, M. G. | 2019 | Of cricket chirps and car horns: The effect of nature sounds on cognitive performance | 10.3758/s13423-018-1539-1 |
| Verheyen, V. J.; Remy, S.; Lambrechts, N.; Govarts, E.; Colles, A.; Poelmans, L.; Verachtert, E.; Lefebvre, W.; Monsieurs, P.; Vanpoucke, C.; Nielsen, F.; van den Eeden, L.; Jacquemyn, Y.; Schoeters, G. | 2021 | Residential exposure to air pollution and access to neighborhood greenspace in relation to hair cortisol concentrations during the second and third trimester of pregnancy | 10.1186/s12940-021-00697-z |
| Vos, S.; Bijnens, E. M.; Renaers, E.; Croons, H.; van der Stukken, C.; Martens, D. S.; Plusquin, M.; Nawrot, T. S. | 2022 | Residential green space is associated with a buffering effect on stress responses during the COVID-19 pandemic in mothers of young children, a prospective study | 10.1016/j.envres.2021.112603 |
| Vujcic, M.; Tomicevic-Dubljevic, J.; Zivojinovic, I.; Toskovic, O. | 2019 | Connection between urban green areas and visitors' physical and mental well-being | 10.1016/j.ufug.2018.01.028 |
| Wallner, P.; Kundi, M.; Arnberger, A.; Eder, R.; Allex, B.; Weitensfelder, L.; Hutter, H. P. | 2018 | Reloading Pupils' Batteries: Impact of Green Spaces on Cognition and Wellbeing | 10.3390/ijerph15061205 |
| Wang, X. X.; Zhou, Q. F.; Zhang, M. J.; Zhang, Q. H. | 2021 | Exercise in the Park or Gym? The Physiological and Mental Responses of Obese People Walking in Different Settings at Different Speeds: A Parallel Group Randomized Trial | 10.3389/fpsyg.2021.728826 |
| Wang, Xiaobo; Shi, Yaxing; Zhang, Bo; Chiang, Yencheng | 2019 | The Influence of Forest Resting Environments on Stress Using Virtual Reality | 10.3390/ijerph16183263 |
| Wang, Y. Q.; Jiang, M. Y.; Huang, Y. S.; Sheng, Z. Y.; Huang, X.; Lin, W.; Chen, Q. B.; Li, X.; Luo, Z. H.; Lv, B. Y. | 2020 | Physiological and Psychological Effects of Watching Videos of Different Durations Showing Urban Bamboo Forests with Varied Structures | 10.3390/ijerph17103434 |
| Wang, Y. Q.; Qu, H. H.; Bai, T.; Chen, Q. B.; Li, X.; Luo, Z. H.; Lv, B. Y.; Jiang, M. Y. | 2021 | Effects of Variations in Color and Organ of Color Expression in Urban Ornamental Bamboo Landscapes on the Physiological and Psychological Responses of College Students | 10.3390/ijerph18031151 |
| Weber, E.; Schneider, I. E. | 2021 | Blooming alleys for better health: Exploring impacts of small-scale greenspaces on neighborhood wellbeing | 10.1016/j.ufug.2020.126950 |
| White, M. P.; Elliott, L. R.; Grellier, J.; Economou, T.; Bell, S.; Bratman, G. N.; Cirach, M.; Gascon, M.; Lima, M. L.; Lohmus, M.; Nieuwenhuijsen, M.; Ojala, A.; Roiko, A.; Schultz, P. W.; van den Bosch, M.; Le Fleming | 2021 | Associations between green/blue spaces and mental health across 18 countries | 10.1038/s41598-021-87675-0 |
| Wiley, E. R.; Stranges, S.; Gilliland, J. A.; Anderson, K. K.; Seabrook, J. A. | 2022 | Residential greenness and substance use among youth and young adults: Associations with alcohol, tobacco, and marijuana use | 10.1016/j.envres.2022.113124 |
| Wolf, L. J.; Ermgassen, S. zu; Balmford, A.; White, M.; Weinstein, N. | 2017 | Is Variety the Spice of Life? An Experimental Investigation into the Effects of Species Richness on Self-Reported Mental Well-Being | 10.1371/journal.pone.0170225 |
| Wood, C. J.; Smyth, N. | 2020 | The health impact of nature exposure and green exercise across the life course: a pilot study | 10.1080/09603123.2019.1593327 |
| Wood, E.; Harsant, A.; Dallimer, M.; Chavez, A. C. de; McEachan, R. R.C.; Hassall, C. | 2018 | Not All Green Space Is Created Equal: Biodiversity Predicts Psychological Restorative Benefits From Urban Green Space | 10.3389/fpsyg.2018.02320 |
| Wood, L.; Hooper, P.; Foster, S.; Bull, F. | 2017 | Public green spaces and positive mental health - investigating the relationship between access, quantity and types of parks and mental wellbeing | 10.1016/j.healthplace.2017.09.002 |
| Wortzel, J. D.; Wiebe, D. J.; DiDomenico, G. E.; Visoki, E.; South, E.; Tam, V.; Greenberg, D. M.; La Brown; Gur, R. C.; Gur, R. E.; Barzilay, R. | 2021 | Association Between Urban Greenspace and Mental Wellbeing During the COVID-19 Pandemic in a US Cohort | 10.3389/frsc.2021.686159 |
| Wu, L. J.; Dong, Q. D.; Luo, S. X.; Jiang, W. Y.; Hao, M.; Chen, Q. B. | 2021 | Effects of Spatial Elements of Urban Landscape Forests on the Restoration Potential and Preference of Adolescents | 10.3390/land10121349 |
| Wu, L. J.; Dong, Q. D.; Luo, S. X.; Li, Y. L.; Liu, Y. Z.; Li, J. N.; Zhu, Z. X.; He, M. L.; Luo, Y. H.; Chen, Q. B. | 2022 | An Empirical Study of the Restoration Potential of Urban Deciduous Forest Space to Youth | 10.3390/ijerph19063453 |
| Wu, W.-H.; Chiou, W.-B. | 2019 | Exposure to pictures of natural landscapes may reduce cigarette smoking | 10.1111/add.14718 |
| Wu, Y. J.; Li, Q. Y.; Zheng, H. Q.; Luo, S. J.; Liu, Q. Y.; Yan, Z.; Huang, Q. T. | 2022 | Factors Influencing Users' Perceived Restoration While Using Treetop Trails: The Case of the Fu and Jinjishan Forest Trails, Fuzhou, China | 10.3390/ijerph19042242 |
| Wu, Y.; Zhuo, Z.; Liu, Q.; Yu, K.; Huang, Q.; Liu, J. | 2021 | The relationships between perceived design intensity, preference, restorativeness and eye movements in designed urban green space | 10.3390/ijerph182010944 |
| Wu, Z. F.; Ren, Y. | 2021 | The influence of greenspace characteristics and building configuration on depression in the elderly | 10.1016/j.buildenv.2020.107477 |
| Wyles, K. J.; White, M. P.; Hattam, C.; Pahl, S.; King, H.; Austen, M. | 2019 | Are Some Natural Environments More Psychologically Beneficial Than Others? The Importance of Type and Quality on Connectedness to Nature and Psychological Restoration | 10.1177/0013916517738312 |
| Xie, B.; Lu, Y.; Zheng, Y. L. | 2022 | Casual evaluation of the effects of a large-scale greenway intervention on physical and mental health: A natural experimental study in China | 10.1016/j.ufug.2021.127419 |
| Xie, Y.; Xiang, H.; Di, N.; Mao, Z.; Hou, J.; Liu, X.; Huo, W.; Yang, B.; Dong, G.; Wang, C.; Chen, G.; Guo, Y.; Li, S. | 2020 | Association between residential greenness and sleep quality in Chinese rural population | 10.1016/j.envint.2020.106100 |
| Xu, J. X.; Wang, F. H.; Chen, L.; Zhang, W. Z. | 2021 | Perceived urban green and residents' health in Beijing | 10.1016/j.ssmph.2021.100790 |
| Yang, H.; Cui, X.; Dijst, M.; Tian, S.; Chen, J.; Huang, J. | 2022 | Association Between Natural/Built Campus Environment and Depression Among Chinese Undergraduates: Multiscale Evidence for the Moderating Role of Socioeconomic Factors After Controlling for Residential Self-Selection | 10.3389/fpubh.2022.844541 |
| Yang, L.; Ho, J. Y.S.; Wong, F. K.Y.; Chang, K. K.P.; Chan, K. L.; Wong, M. S.; Ho, H. C.; Yuen, J. W.M.; Huang, J. X.; Siu, J. Y.M. | 2020 | Neighbourhood green space, perceived stress and sleep quality in an urban population | 10.1016/j.ufug.2020.126763 |
| Yang, M.; Dijst, M.; Faber, J.; Helbich, M. | 2020 | Using structural equation modeling to examine pathways between perceived residential green space and mental health among internal migrants in China | 10.1016/j.envres.2020.109121 |
| Yang, T. Z.; Barnett, R.; Fan, Y. F.; Li, L. | 2019 | The effect of urban green space on uncertainty stress and life stress: A nationwide study of university students in China | 10.1016/j.healthplace.2019.102199 |
| Yang, Y.; Wang, L. S.; Passmore, H. A.; Zhang, J.; Zhu, L. F.; Cai, H. J. | 2021 | Viewing nature scenes reduces the pain of social ostracism | 10.1080/00224545.2020.1784826 |
| Yigitcanlar, T.; Kamruzzaman, M.; Teimouri, R.; Degirmenci, K.; Alanjagh, F. A. | 2020 | Association between park visits and mental health in a developing country context: The case of Tabriz, Iran | 10.1016/j.landurbplan.2020.103805 |
| Yin, J.; Arfaei, N.; MacNaughton, P.; Catalano, P. J.; Allen, J. G.; Spengler, J. D. | 2019 | Effects of biophilic interventions in office on stress reaction and cognitive function: A randomized crossover study in virtual reality | 10.1111/ina.12593 |
| Yin, Y. T.; Shao, Y. H.; Xue, Z. Y.; Thwaites, K.; Zhang, K. X. | 2020 | AN EXPLORATIVE STUDY ON THE IDENTIFICATION AND EVALUATION OF RESTORATIVE STREETSCAPE ELEMENTS | 10.15302/J-LAF-0-020005 |
| Yoo, E. H.; Roberts, J. E.; Eum, Y.; Li, X. J.; Konty, K. | 2022 | Exposure to urban green space may both promote and harm mental health in socially vulnerable neighborhoods: A neighborhood-scale analysis in New York City | 10.1016/j.envres.2021.112292 |
| Yoshino, Aiko; Wilson, Jackson; Velazquez, Edgar J.; Johnson, Eric; Márquez-Magaña, Leticia | 2018 | Healthy Parks Healthy People as an Upstream Stress Reduction Strategy | 10.2979/rptph.2.1.03 |
| Young, C.; Hofmann, M.; Frey, D.; Moretti, M.; Bauer, N. | 2020 | Psychological restoration in urban gardens related to garden type, biodiversity and garden-related stress | 10.1016/j.landurbplan.2020.103777 |
| Yu, C. P.; Lee, H. Y.; Lu, W. H.; Huang, Y. C.; Browning, MHEM | 2020 | Restorative effects of virtual natural settings on middle-aged and elderly adults | 10.1016/j.ufug.2020.126863 |
| Yu, C. P.; Lee, H. Y.; Luo, X. Y. | 2018 | The effect of virtual reality forest and urban environments on physiological and psychological responses | 10.1016/j.ufug.2018.08.013 |
| Zabini, F.; Albanese, L.; Becheri, F. R.; Gavazzi, G.; Giganti, F.; Giovanelli, F.; Gronchi, G.; Guazzini, A.; Laurino, M.; Li, Q.; Marzi, T.; Mastorci, F.; Meneguzzo, F.; Righi, S.; Viggiano, M. P. | 2020 | Comparative Study of the Restorative Effects of Forest and Urban Videos during COVID-19 Lockdown: Intrinsic and Benchmark Values | 10.3390/ijerph17218011 |
| Zayas-Costa, M.; Cole, H. V.S.; Anguelovski, I.; Connolly, J. J.T.; Bartoll, X.; Triguero-Mas, M. | 2021 | Mental Health Outcomes in Barcelona: The Interplay between Gentrification and Greenspace | 10.3390/ijerph18179314 |
| Zhang, C.; Wang, C.; Chen, C.; Tao, L. Y.; Jin, J. L.; Wang, Z. Y.; Jia, B. Q. | 2022 | Effects of tree canopy on psychological distress: A repeated cross-sectional study before and during the COVID-19 epidemic | 10.1016/j.envres.2021.111795 |
| Zhang, J.; Yang, Z.; Chen, Z.; Guo, M. Y.; Guo, P. | 2021 | Optimizing Urban Forest Landscape for Better Perceptions of Positive Emotions | 10.3390/f12121691 |
| Zhang, L. Q.; Tan, P. Y.; Richards, D. | 2021 | Relative importance of quantitative and qualitative aspects of urban green spaces in promoting health | 10.1016/j.lurbplan.2021.104131 |
| Zhang, L.; Liu, S. Y.; Liu, S. | 2021 | Mechanisms Underlying the Effects of Landscape Features of Urban Community Parks on Health-Related Feelings of Users | 10.3390/ijerph18157888 |
| Zhang, Lingling; Luo, Ye; Zhang, Yao; Pan, Xi; Zhao, Dandan; Wang, Qing | 2022 | Green Space, Air Pollution, Weather, and Cognitive Function in Middle and Old Age in China | 10.3389/fpubh.2022.871104 |
| Zhang, X.; Wei, F.; Yu, Z.; Guo, F.; Wang, J.; Jin, M.; Shui, L.; Lin, H.; Tang, M.; Chen, K. | 2022 | Association of residential greenness and incident depression: Investigating the mediation and interaction effects of particulate matter | 10.1016/j.scitotenv.2021.152372 |
| Zhang, Y.; Kang, J. | 2017 | Effects of Soundscape on the Environmental Restoration in Urban Natural Environments | 10.4103/nah.NAH_73_16 |
| Zhao, S.; Patuano, A. | 2022 | International Chinese Students in the UK: Association between Use of Green Spaces and Lower Stress Levels | 10.3390/su14010089 |
| Zhou, R.; Zheng, Y.-J.; Yun, J.-Y.; Wang, H.-M. | 2022 | The Effects of Urban Green Space on Depressive Symptoms of Mid-Aged and Elderly Urban Residents in China: Evidence from the China Health and Retirement Longitudinal Study | 10.3390/ijerph19020717 |
| Zhou, Y. Q.; Yuan, Y.; Chen, Y. J.; Lai, S. L. | 2020 | Association Pathways Between Neighborhood Greenspaces and the Physical and Mental Health of Older Adults-A Cross-Sectional Study in Guangzhou, China | 10.3389/fpubh.2020.551453 |
| Zhu, an; Yan, L. J.; Shu, C.; Zeng, Y.; Ji, J. S. | 2020 | APOE epsilon 4 Modifies Effect of Residential Greenness on Cognitive Function among Older Adults: A Longitudinal Analysis in China | 10.1038/s41598-019-57082-7 |
| Zhu, Anna; Wu, Chenkai; Yan, Lijing L.; Wu, Chih-Da; Bai, Chen; Shi, Xiaoming; Zeng, Yi; Ji, John S. | 2019 | Association between residential greenness and cognitive function: analysis of the Chinese Longitudinal Healthy Longevity Survey | 10.1136/bmjnph-2019-000030 |
| Zijlema, W. L.; Avila-Palencia, I.; Triguero-Mas, M.; Gidlow, C.; Maas, J.; Kruize, H.; Andrusaityte, S.; Grazuleviciene, R.; Nieuwenhuijsen, M. J. | 2018 | Active commuting through natural environments is associated with better mental health: Results from the PHENOTYPE project | 10.1016/j.envint.2018.10.002 |
| Zijlema, W. L.; Triguero-Mas, M.; Smith, G.; Cirach, M.; Martinez, D.; Dadvand, P.; Gascon, M.; Jones, M.; Gidlow, C.; Hurst, G.; Masterson, D.; Ellis, N.; van den Berg, M.; Maas, J.; van Kamp, I.; van den Hazel, P.; Kruize, H.; Nieuwenhuijsen, M. J.; Julvez, J. | 2017 | The relationship between natural outdoor environments and cognitive functioning and its mediators | 10.1016/j.envres.2017.02.017 |
